# A Comprehensive Review of the Current Progress of Chromium Removal Methods from Aqueous Solution

**DOI:** 10.3390/toxics11030252

**Published:** 2023-03-08

**Authors:** Md. Monjurul Islam, Anika Amir Mohana, Md. Aminur Rahman, Mahbubur Rahman, Ravi Naidu, Mohammad Mahmudur Rahman

**Affiliations:** 1 Applied Chemistry and Chemical Engineering, Faculty of Engineering and Technology, Islamic University, Kushtia 7003, Bangladesh; 2 Global Centre for Environmental Remediation (GCER), College of Engineering, Science and Environment, The University of Newcastle, Callaghan, NSW 2308, Australia; 3 Zonal Laboratory, Department of Public Health Engineering (DPHE), Jashore 7400, Bangladesh; 4 Chittagong University of Engineering and Technology, Faculty of Civil Engineering, Chattogram 4349, Bangladesh; 5 CRC for Contamination Assessment and Remediation of the Environment, The University of Newcastle, Callaghan, NSW 2308, Australia; 6 Department of General Educational Development, Faculty of Science & Information Technology, Daffodil International University, Dhaka 1207, Bangladesh

**Keywords:** chromium, adsorption, remediation, wastewater

## Abstract

Chromium (Cr) exists in aqueous solution as trivalent (Cr^3+^) and hexavalent (Cr^6+^) forms. Cr^3+^ is an essential trace element while Cr^6+^ is a dangerous and carcinogenic element, which is of great concern globally due to its extensive applications in various industrial processes such as textiles, manufacturing of inks, dyes, paints, and pigments, electroplating, stainless steel, leather, tanning, and wood preservation, among others. Cr^3+^ in wastewater can be transformed into Cr^6+^ when it enters the environment. Therefore, research on Cr remediation from water has attracted much attention recently. A number of methods such as adsorption, electrochemical treatment, physico-chemical methods, biological removal, and membrane filtration have been devised for efficient Cr removal from water. This review comprehensively demonstrated the Cr removal technologies in the literature to date. The advantages and disadvantages of Cr removal methods were also described. Future research directions are suggested and provide the application of adsorbents for Cr removal from waters.

## 1. Introduction

**Figure 1:**
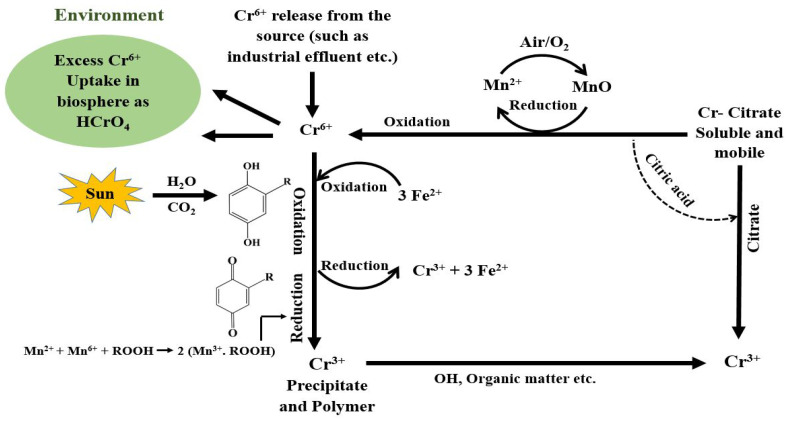
Chromium cycle in the environment.

Chromium (Cr) is classified as a Group 1 element that is carcinogenic to living organisms, is a great risk to the environment, and is ranked fifth of the potentially worst toxic elements [1]. The superfund-controlled contaminated sites in the United States declared Cr as one of the top 20 toxic substances [2]. Most water sources contain only the hexavalent (Cr^6+^) and trivalent (Cr^3+^) forms of Cr in stable states [3]. Compounds of Cr^3+^ are adsorbed and form precipitates with very low solubility, which hinders its leaching into groundwater. Comparatively, Cr^6+^ exhibits high stability and strong oxidation [4,5]. Thus, due to its non-biodegradability behavior, Cr^6+^ has the potential to be hidden and persist in the long-term [6,7]. Cr is mainly used for the production of Cr-based alloys (around 60%) while the rest is consumed for electroplating, furnace blocks, and other refractory items [8,9]. The Cr metal contains a body-centered cubic crystal system which makes Cr an appropriate component that can improve compounds and increment consumption resistivity, color change, metallic luster, and additionally hardness [10]. The life circle of Cr in the environment is depicted in Figure 1.

The conveyance of compounds containing Cr^3+^ and Cr^6+^ relies upon redox potential, pH, presence of oxidizing or reducing compounds, the kinetics of the redox reactions, formation of Cr^3+^ complexes or insoluble Cr^3+^ salts, and total Cr concentration [11]. In general, Cr^6+^ salts are more soluble than those of Cr^3+^, making Cr^6+^ moderately portable [12].

**Figure 2:**
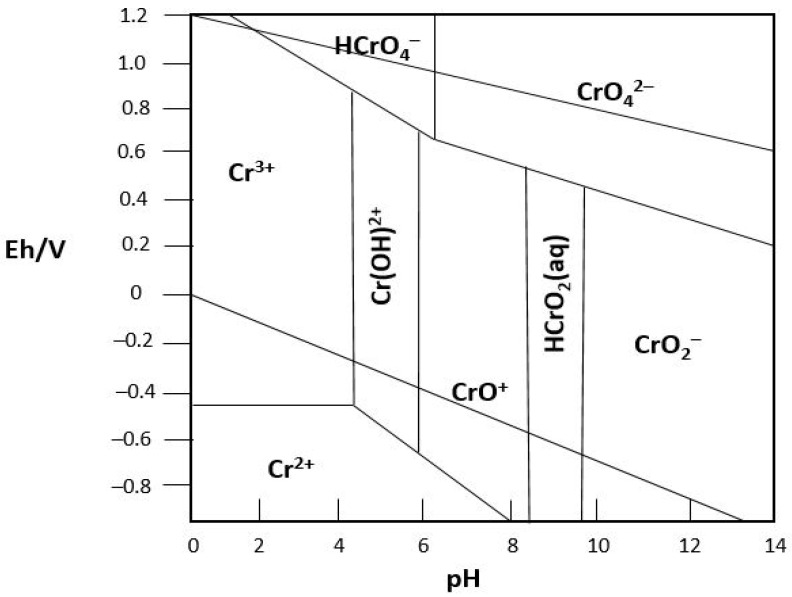
Pourbaix diagram for Cr chemical species in aqueous solution (Chen et al. 2021) [13].

The major forms of Cr^6+^ in an aqueous solution are Cr_2_O_7_^2−^, CrO_4_^2−^, H_2_CrO_4_, and HCrO_4_^−^ [13]. The pH of the solution, the overall concentration of Cr, the presence of oxidizing and reducing chemicals, the redox potential, and the kinetics of redox processes all affect how this distribution is distributed. Whereas HCrO_4_^−^ predominates in the pH range of one to six, CrO_4_^2−^ is the only ion present if the solution’s pH is higher than 7 (Figure 2). When the pH is below 3.9, Cr^3+^ exists as water-soluble Cr^3+^ cations, and as the pH rises to 5, the amount of Cr^3+^ steadily declines (Figure 2). Hydrolysis produces Cr(OH)^2+^ when pH is greater than 5. Water-insoluble Cr(OH)_3_ precipitate forms when the pH is greater than 6. Compounds containing Cr^3+^ are easily absorbed by soil colloids and create deposits with a very low solubility, which prevents them from penetrating the groundwater or being taken up by plants. The chromate and dichromate forms of Cr^6+^ (CrO_4_^2−^, HCrO_4_^−^, and Cr_2_O_7_^2−^), on the other hand, have strong oxidative and high solubility properties [14].

The solubility of Cr^3+^ is minimum in natural water at pH 7.5–8.5. Cr^6+^ exists in solution as monomeric species/ions: H_2_CrO_4_^0^, HCrO_4_^−^ (hydrogen chromate), and CrO_4_^2−^ (chromate); or as the dimeric ion Cr_2_O_7_^2−^ (dichromate, exists in emphatically acidic solution) [11]. At pH 1–10 and low concentration, Cr exists as either monovalent HCrO_4_^−^ or divalent chromate CrO_4_^2−^ in groundwater. The monovalent structure prevails in acidic water while the divalent structure prevails at neutral pH or above. The monomeric species of Cr^6+^ with a concentration of more than 1 mg/L is responsible for the yellow color of water [11]. As indicated by the World Health Organization (WHO) drinking water guidelines, the greatest permissible cutoff for Cr is 0.05 mg/L [15].

**Figure 3:**
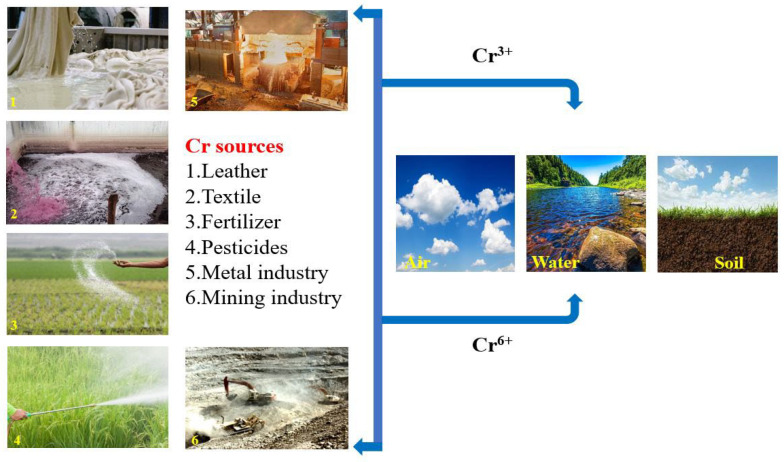
Exposure of chromium.

Normally, Cr concentrations in groundwater are very low (below 2 mg/L), in spite of the fact that concentrations as high as 120 mg/L have been revealed (WHO 2006). It enters into various environmental systems (air, water, soil, etc.), through some natural processes (mining of ores) and anthropogenic activities such as refining and mining of ores, pesticides, batteries, paper industries, tanneries, fertilizer applications, and solid wastes disposal including sewage sludge, wastewater irrigation, and vehicular exhaust [4,16,17,18,19,20]. Figure 3 shows how toxic Cr emerges from various sources in our environment.

Adsorption of Cr^6+^ is normally restricted and diminishes with expanding pH [9,21]. Fe^2+^, certain organic compounds with sulfhydryl groups, and sulfides can reduce Cr^6+^ effectively. Interestingly, Cr^3+^ is oxidized quickly by an enormous excess of MnO_2_ and gradually by oxygen under conditions such as natural waters. It can therefore be asserted that both the concentration of Cr^6+^ and absolute Cr define the quality of water. Indeed, even at Cr^6+^ levels estimated in the parts per billion (ppb), it appears to be poisonous [22]. Cr^6+^ can enter or infiltrate the cell divider and apply its poisonous impact in the cell itself, serving as a wellspring of different malignant growth sicknesses or diseases [23,24,25]. At transient exposure levels above the most extreme contaminant level, Cr^6+^ causes skin and stomach disturbance or ulceration.

Long-haul introduction at levels above the greatest contaminant can cause dermatitis, harm to the liver, kidney dissemination, nerve tissue harm, and death as a result of huge dosages [26,27]. Cr^6+^ is substantially more toxic than Cr^3+^ for both intense and interminable exposures [15,28]. Acute ingestion of large amounts of Cr^6+^ causes gastrointestinal problems, including stomach torment, vomiting, and hemorrhage, while ceaseless exposure to Cr^6+^ brings about perforations and ulcerations of the septum, which have dangerous consequences for the respiratory tract. Epidemiological and animal studies recommend that Cr^6+^ mixtures, especially those that are water-insoluble, are responsible for different types of DNA harm and carcinogenicity [29,30]. Consequently, urgent and efficient Cr clean-up technology is essential.

Considering the above Cr toxicity in the environment, a number of different methods have been used for several years to remove Cr from different wastewaters, such as adsorption, electrochemical treatments, physico-chemical processes, biological removal (phytoremediation), membrane filtration, and chelation. Several articles have discussed the removal technologies of Cr. Notably, Aigbe and Osibote (2020) discussed hexavalent chromium removal from aqueous solutions utilizing the sorption technique with nanomaterials [31]. Another review paper published by Ukhurebor et al. (2021) discussed the effects and remediation of hexavalent Cr from soil [25]. Sharma et al. (2008) focused their review entirely on various technologies that remove Cr from water [11]. These authors discussed five different technologies that had been used till 2008 for removing Cr from wastewater [32]. Another review article published by Narayani and Shetty (2013) discussed the use of chromium-resistant bacteria for the removal of hexavalent Cr [33]. However, there is a significant paucity of research on the overall Cr removal techniques (both Cr^3+^ and Cr^6+^) in recent years. The present review paper comprehensively examines various techniques employed for the removal of Cr, expatiating upon a multitude of experimental parameters. Notably, a meticulous comparison of the advantages and disadvantages inherent to each technique is provided. Additionally, this review presents an insightful discussion concerning the novel research directions and promising future prospects, thus contributing significantly to the advancement of knowledge in this field.

## 2. Cr Removal Methods

There are various methods available in the literature for the removal of Cr from wastewater, which are mostly suitable for eliminating high concentrations of the substance. Traditional removal methods usually focus on removing and reusing Cr [32]. Conventional methods that have been employed in Cr removal include chemical reduction, precipitation, flotation, solvent extraction, membrane separation (ultrafiltration, reverse osmosis, nanofiltration, microfiltration, hybrid membrane systems, ion exchange membranes, surfactant-based membranes, and liquid membranes), electrochemical treatment (electrodialysis, electrolysis, electrocoagulation) and ion exchange [25]. Of these, flocculation and membrane separation are very popular for industrial applications. Currently, the most important technology for Cr removal from wastewater is chemical precipitation. However, biosorption and metal oxide adsorption are cost-effective and promote recyclability [31]. The advantages and disadvantages of these methods are shown in Table 1.

### 2.1. Adsorption

Nowadays adsorption is considered to be a suitable and economical technique for the removal of Cr from wastewater. In this process, Cr ion adheres to the surface of the adsorbent that has high surface area due to its porosity. Surface charge of the adsorbent plays an important role in adsorption mechanism. Modification of surface charge by changing the functional group can enhance the capacity of adsorbent. Among various modification methods, nitrogenation, oxidation, and sulfuration are the most employed techniques to enhance the specific surface area, pore structure, adsorption capacity, thermal stability, and mechanical strength [47]. However, they depend mainly on adsorbent materials, which sometimes are very expensive. Subsequently, the cost of adsorbent should be considered in choosing the most suitable adsorbents [48]. In light of the fact that adsorption is sometimes reversible, adsorbents can be recovered by reasonable desorption. In comparison with conventional methods, adsorption has various advantages such as minimal cost, availability, ease of operation, and efficiency, especially from economic and environmental points of view [49,50,51]. There are various suitable adsorbents available including natural adsorbents, composites, bio-sorbents, metal oxides, etc., for the removal of Cr.

#### 2.1.1. Natural Adsorbents

Various materials such as natural materials, agricultural wastes, etc., can be deployed as low-cost adsorbents. It is found to be much more encouraging to use these adsorbents for the removal of heavy metals [52]. These low-cost adsorbents can be collected from natural and anthropogenic sources. Different kinds of natural adsorbents are being used in recent times such as pulp [53], clay [54,55], clinoptilolite [56], zeolite [57], leaf [58], activated carbon [59], charcoal [60], green walnut shell [61], etc. (Table 2).

Some research found that clay minerals are economical as adsorbents for removing industrial pollutants. Zhao et al. (2015) used pristine Akadama clay (AC) activated by HCl [55]. The experiments were conducted using the batch process. The optimum pH ranged between 2 and 3–9 after the activation of the clay (HCl–AC). The pseudo-second-order model serves to explain the kinetics of the obtained experimental data. Around 98.9% of Cr^6+^ was removed from tannery wastewater using the mentioned adsorbent, which can easily compete with other adsorbents. In their work, Bentchikou et al. (2017) investigated the removal of Cr from aqueous solution, using natural Algerian brown clay, in batch mode, at different temperatures [54]. Kinetic experiments showed that the pseudo-second-order model can explain the adsorption process.

The results showed that natural brown clay is effective in removing about 90% of Cr^6+^ at 20 °C and contact time was 60 min. At that same period of time, Zanin et al. (2017) reported natural clinoptilolite zeolite as an adsorbent could remove Cr^3+^ in wastewater from the graphic industry [56]. These authors found that up to 85% Cr removal is possible at 25 °C and pH 4.0. Elsewhere, Adam et al. (2018) used both natural zeolite and clinoptilolite, in the form of hollow fiber ceramic membrane (HFCM) to remove Cr^6+^ from aqueous solution [57]. The performance of the HFCM in adsorption/filtration was 44% of Cr^6+^ removal at the initial concentration of 40 mg/L and pH 4.

Dehghani et al. (2016) used treated waste newspaper pulp (TWNP) to remove Cr^6+^ from aqueous solution using batch experiments [53]. The adsorption parameters were: initial Cr^6+^ concentration (5, 20, 50 mg/L), contact time (60 min), adsorbent dose (3.0 g/L), and solution pH (3.0). The adsorption of Cr^6+^ was pH dependent and the experimental data fitted well to the Langmuir isotherm (R^2^ = 0.98; maximum adsorption capacity 59.88 mg/g) and pseudo-second-order kinetics model. The rate constant k_2_ varied from 0.0019 to 0.0068 at an initial Cr^6+^ concentration ranging from 5 to 20 mg/L. The percentage of Cr^6+^ removed was 59.88 mg/g (64% at pH 3). Nag et al. (2016) prepared both batch and continuous column mode experiments for rubber leaf and the pseudo-second-order model firmly described the kinetic process with a correlation coefficient of 0.99 [58].

Cr^6+^ (100%) was spontaneously removed according to an endothermic process at pH 1.5. Yasmeen et al. (2016) prepared synthesized adsorbent from shrimp shells and waste cotton rags which proved to be a viable adsorbent for removing Cr^6+^ ions from tannery effluent. Here the optimum pH for the maximum adsorption was pH 5.0, 93% with a composite concentration (2 g/L) and the effective rate was 240 min [138]. It emerged that Langmuir isotherm was well fitted with R^2^ value of 0.997. Wan et al. (2018) tested m-phenylenediamine-modified magnetic chitosan for the concurrent reduction-absorption of Cr^6+^ and 227.27 mg/g Cr could be removed at pH < 4 [137]. Wang et al. (2019) used a β-cyclodextrin (b-CD) functionalized three-dimensional structured graphene foam for the removal of Cr^6+^ and achieved 99.8% efficiency at pH 3 [136].

Activated carbon can be another feasible solution for Cr removal from water. Agarwal and Gupta (2015) used animal bone charcoal (ABC) as an adsorbent to remove Cr^6+^ from effluent water at pH 2 with an initial concentration of 100 mg/L at constant temperature [60]. After shaking for 120 min at 140 rpm, 92% was removed. In their research, Zafarani et al. (2015) investigated the adsorption capacity of green walnut shell (GWS) for the removal of Cr^6+^ remaining in aqueous medium using batch experiments [61]. The ideal operational conditions for Cr^6+^ removal was: 10 mg/L, pH = 3.6, t = 5 min, and GWS doses = 6 g/L. The corresponding Cr^6+^ removal efficiency was 95%. In a similar study, Gottipati and Mishra (2016) developed microporous activated carbon (MAC) from an economically feasible plant precursor, i.e., *Aegle marmelos* fruit shell using ZnCl_2_ activation and 82.3% removal of Cr^6+^ became possible at pH 2.0 [59].

Li et al. (2020) investigated the removal of Cr from aqueous solution using Zn- and Al-modified pristine hydrochar. Kinetic experiments showed that the pseudo-second-order model could explain the adsorption process and fit the Langmuir model [135]. The results showed that this modified hydrochar is effective in removing Cr^6+^ at pH 2–4. Qiao et al. (2020) prepared floatable magnetic iron/biochar beads (FMIB) using *Enteromorpha prolifera,* where the optimum ratio was 2:1, for the removal of Cr species [134]. After three cycles, the FMIB bed successfully removed 21.5% and 40.5% of Cr^6+^ and Cr^3+^, respectively. Furthermore, the experiments show that it has the potential to remove 87.7% of the total Cr from leather processing wastewater. The best level of pH for this process was 4. The contact times for Cr^3+^ and Cr^6+^ were 120–480 min and 900 min, respectively. This result demonstrated the kinetics could be described using the pseudo-second-order model and the isotherm model fitted the Langmuir model well. Recently, Jabłońska (2020) used shale waste rocks to remove Cr^3+^ and Cr^6+^ from aqueous solution [133]. The maximum sorption (90–91%) took place at pH 3–6 for Cr^3+^ and pH 4–5 for Cr^6+^. The sorption process was well described by the Langmuir–Freundlich isotherm model. Though natural adsorbents are good for the adsorption of Cr, they are blended to form a composite to improve efficiency.

#### 2.1.2. Bio-Sorbents

Each type of biological species has an attraction for metals, yet microbial organisms such as bacteria and fungi are prudently used to execute most of the biosorption experiments [139,140]. Many researchers in removing Cr used bio-materials instead of available physico-chemical advances as a dynamic alternative. Several reviews have been published on the usage of various kinds of bio-sorbents, which are famous for their Cr binding capacities, for example, agricultural wastes, bacteria, fungi, algae, etc. [139,140,141,142,143,144,145].

##### Bacteria

To remove Cr^3+^ and Cr^6+^ from wastewater, several types of bacteria have been studied, for example: *Spirulina* sp. [111], *Escherichia coli* [112], *Staphylococcus epidermidis* [112], *Rhodococcus opacus* [113], *Rhodococcus rhodochrou* [113], *Staphylococcus* sp. [114], *Pseudomonas* sp. [114], *Azotobacter beijreinckii* [115], and *Bacillus subtilis* [115].

The experiment carried out by Rezaei (2016) using dried *Spirulina* sp. biomass showed that at 40 °C and pH 5, the biomass confirmed the highest Cr^6+^ adsorption capacity when the initial concentration was 10 mg/L [111]. The Freundlich model fitted experimental data better than the Langmuir model as both models were used to analyze them and maximum biosorption capacity was found to be 90.91 mg/g. In their paper, Quiton et al. (2018) reported *E. coli* and *Staphylococcus epidermidis* biofilms supported on kaolin for the removal of Cr^6+^ from aqueous solution [112] (Table 2). Optimum pH for the biosorption of Cr^6+^ was 3.0–6.0. When the initial concentration of Cr was 200 mg/L, the highest biosorption capacity was 16.9 mg/g. The biosorption of Cr^6+^ by *E. coli* biofilm supported on kaolin fits the Langmuir isotherm well, whereas the Freundlich isotherm best describes the Cr^6+^ biosorption onto *S. epidermidis* biofilm.

On the other hand, Dobrowolski et al. (2017) chose two different kinds of species of bacteria, namely *Rhodococcus opacus* and *Rhodococcus rhodochrous* and carried out the experiment with pH 5 at 25 °C in batch process for the removal of Cr^6+^ from aqueous solution [113]. The equilibrium data of the biosorption was well described by the Freundlich isotherm model rather than the Langmuir model. The maximum removals of Cr^6+^ were 82 and 62 mg/g for biopolymer from *Rhodococcus rhodochrous* and *Rhodococcus opacus,* respectively, which are very low. Thus, these bio-sorbents are not suitable for Cr^6+^ removal. Emran et al. (2019) also used two different kinds of species—*Staphylococcus* sp. and *Pseudomonas* sp.—for the biosorption of Cr^6+^ and determined the optimum pH, contact time, and biomass concentration [114]. The equilibrium time was 24 h when the desired pH was 5 at 50 °C. The equilibrium data were well described by both the Langmuir and Freundlich isotherms. Meanwhile, Chug et al. (2016) used both *Azotobacter beijreinckii* and *Bacillus subtilis* bacteria for developing Extracellular Polymeric Substances to remove Cr^6+^ [115]. *A. beijreinckii* and *B. subtilis* bacteria could remove 26% and 48% of Cr^6+^, respectively, from aqueous solution after 24 h incubation. This occurred when the initial concentration of Cr was 10 mg/L. After 24 h, the removal percentage is independent of incubation period (Table 2).

##### Fungi

One of the best-known low-cost adsorbents for removing Cr from wastewater is fungi biomass. To date, *Aspergillus niger* was found to be the most efficient fungi [102,104]. Saravanan et al. (2016) [102] and Mondal et al. (2017) [101] used blended *Aspergillus niger.* Saravanan et al. (2016) [102] used these fungi with custard apple seeds for experiments that were carried out in the batch process (Table 2). Authors found that the optimum initial concentration was 100 mg/L and bio-sorbent loading was 10 g/L when the pH was 3.0 at 36 °C. The largest amount of Cr^6+^ removed from the wastewater, at the stated conditions, was 95.7%. Mondal et al. (2017) used the batch process for their experiment [101], and these authors found that the optimum values were: initial concentration = 33.33 mg/L, pH = 4.6, adsorbent dose = 1.0 g/L and contact time = 48.45 min. At initial pH of 2.0, the maximum adsorption capacity for *A. niger* was 11.79 mg/g. The biosorption process followed the pseudo-second-order kinetic model, and the equilibrium data were well described by both Temkin and Freundlich isotherms. The activation energy of the adsorption was estimated as 2.9 × 10^−3^ kJ/mol.

Chen et al. (2019) examined the consumption of marine-derived fungus *Penicillium janthinellum* (P1) for the removal of aqueous Cr^6+^ in batch experiments [100]. Their results showed that maximum Cr^6+^ biosorption capacity for living fungus P1 pellets was about 87% at the optimum condition of beginning concentration of Cr^6+^ = 250 mg/L, beginning pH = 1, temperature = 30 °C, bio-sorbent dosage = 30 g/L and contact time = 8 h. Similarly, maximum Cr^6+^ biosorption capacity for non-living fungus P1 pellets amounted to approximately 58.6% at the desired conditions of: initial concentration of Cr^6+^ = 100 mg/L; initial pH = 1; temperature = 30 °C; bio-sorbent dose = 3 g/L; and contact time = 12 h. After analyzing the data, it emerged that the biosorption process was well described by the Freundlich, Dubinin–Radushkevich (D–R) isotherms followed by the pseudo-second-order kinetic model.

In comparison, Mahmoud et al. (2015) produced three highly effective bio-sorbents, i.e., (1) NSi-Asp, (2) NSi-Fus, and (3) NSi-Pen from three fungal strains, named (1) *Aspergillus ustus* (Asp), (2) *Fusarium verticillioides* (Fus), and (3) *Pencillium funiculosum* (Pen),. This was completed by immobilizing them on nanosilica (NSi) surface [103]. The highest biosorption capacities for Cr^3+^ were 2466, 2666, and 1866 μmol/g by using NSi-Asp, NSi-Fus, and NSi-Pen, respectively, at pH 7.0 whereas for Cr^6+^ the values were 6466, 6400, and 3800 μmol/g at pH 2.0. Both Langmuir and Freundlich isotherms were used to describe the data of sorption equilibria, which was obtained at about 15 min. Sivakumar (2016) carried out a comparative investigation of the efficiencies of *Aspergillus foetidus*, *Aspergillus fumigatus*, *Aspergillus heteromorphus*, *Aspergillus viridinutans*, *Aspergillus flavus*, *Aspergillus nidulans*, and *Aspergillus niger* for the removal of Cr^6+^ from tannery wastewater [104]. The outcome was that the sequence for the highest removal of Cr^6+^ with initial concentration of 18.125 mg/L (dilution ratio 4) was: *A. niger* > *A. flavus* > *A. fumigatus* > *A. nidulans* > *A. heteromorphus* > *A. foetidus* > *A. viridinutans* at pH 3 when the fungi biomass was 4 g. Furthermore, *Aspergillus niger* proved to be the most efficient Cr^6+^ remover (96.3%) in comparison with other fungi species when the concentration of Cr^6+^ was kept at 18.12 mg/L.

##### Algae

Several algae species have been used as bio-sorbents for the recovery of Cr from industrial effluents. These include *Scenedesmus* sp. [90], *Scenedesmus quadri-cauda* [91], *Sargassum myriocystum* [92], *Cladophora glomerata* [93], etc. Jayakumar et al. (2015) [92] used *Sargassum myriocystum* as a bio-sorbent. The ideal conditions of the process were: sorbent dosage = 2.017 g/L, contact time = 108 min, pH = 5.2, agitation speed = 120 rpm. The sorption process was well fitted to the Langmuir and Toth isotherm models (R^2^ = 0.993 and 0.992), followed by both pseudo-second-order and Elovich and power function kinetic models, with the highest sorption capacity of 66.66 mg/g. A Chlorophyta named *Scenedesmus quadri-cauda* served as an effective bio-sorbent by Shokri Khoubestani et al. (2015) in order to remove both Cr^3+^ and Cr^6+^ using batch experiments. A total of 98.3% (at pH 6) and 47.6% (at pH 1) of Cr^3+^ and Cr^6+^ were, respectively, removed by this process having an equilibrium time of 120 min [91]. The Langmuir model described the biosorption process of Cr^3+^, while conversely, Freundlich isotherm model was used for Cr^6+^. Both processes followed the pseudo-second-order kinetic model. The maximum biosorption capacities were 58.47 and 46.51 mg/g for Cr^3+^ and Cr^6+^, respectively, according to the Langmuir model (Table 2).

Al-Homaidan et al. (2018) treated *Cladophora glomerata* with acid for the preparation of the bio-sorbent utilized for the removal of heavy metals [93]. The maximum removal of Cr was 66.6% at pH 2.0 when 1.0 g dried algal cells/100 mL aqueous solution containing an initial concentration of 20 mg/L Cr was employed (Table 2). The wastewater and bio-sorbent were kept in contact for 60 min at 45 °C. Although both Langmuir and Freundlich isotherm models can be used to describe the equilibrium data, the Freundlich model fits well. Losada et al. (2018) investigated the removal of Cr^6+^ from tannery wastewater using *Scenedesmus sp* [90]. The initial concentration of Cr was 352.2 mg/L. The result strongly suggested that the greatest Cr removal occurred with 98.63% (4.82 mg/L) effectiveness, acting as a removal agent at pH of 6.0, constant aeration, temperature of 28 °C, and time lasting 48 h. Gupta et al. (2008) conducted sorption and desorption studies of Cr^6+^ from wastewater using nonviable cyanobacterium *Nostoc muscorum* biomass [146]. The maximum Cr^6+^ biosorption capacity for *N. muscorum* has been found to be 22.92 mg/g at a dose of 1.0 g/L with initial Cr^6+^ concentration of 100 mg/L and optimum pH of 3.0. It was found that of all the desorbents tested, EDTA and HNO_3_ were the most effective, whereas desorption with deionized water was essentially negligible [146].

##### Plants

Cr biosorption was examined through the usage of various plants, including *Tradescantia pallida* [86] and *Caryota urens* [87]. Sinha et al. (2015) used *Tradescanti pallida* leaf as a bio-sorbent to remove 94% of Cr^6+^ with a sorption capacity of 64.672 mg/g by batch experiments at pH 2 [86]. Pseudo-second-order kinetic model was used to explain the kinetics of Cr^6+^ while the Langmuir model described the isotherm data better than the Freundlich model. In another study, Suganya et al. (2016) used *Caryota urens* seeds as part of an investigation into the effects of various parameters, such as initial concentration, pH, bio-sorbent dosage, and contact time, on the biosorption of Cr^6+^ (Table 2) [87]. The biosorption process can be described using both Langmuir and Freundlich isotherm models, whereas the kinetic data followed the pseudo-second-order kinetic model. According to the Langmuir isotherm model, the best biosorption capacity was 52.63 mg/g at an optimum pH of 2 when the temperature remained at 303 K. Lin et al. (2018) used aminated rice straw-grafted-poly (vinyl alcohol) (A-RS/PVA) for the removal of Cr^6+^ [88]. From batch adsorption experiments, it can be said that the adsorption data can be described well using both Elovich and Freundlich isotherm models. At initial pH 2.0 and 60 °C the adsorption capacity of A-RS/PVA (140.39 mg/g) was much larger than that of original rice straw (34.9 mg/g).

##### Agricultural Wastes

Agricultural waste is one of the feasible and popular materials for the removal of Cr from wastewater. Ali et al. (2016) used acrylonitrile-grafted banana peels (GBPs) for the removal of Cr^6+^ from wastewater [73]. During the process, grafted banana peels (GBPs) successfully adsorbed 96% of Cr^6+^, when the optimum conditions were pH 3, adsorbent dose 4 g/L, concentration 400 mg/L, and contact time of 120 min (Table 2). Both Freundlich and Langmuir isotherm models were used to describe the adsorption data followed by the pseudo-second-order kinetic model, and it proved to be an exothermic spontaneous process. In their work, Yari et al. (2016) removed hexavalent Cr using rice husk [74]. With biomass dose of 5 g/L, the maximum adsorption capacity was 38.4 mg/g at optimum conditions of pH 5.0 and contact time of 75 min at 30 °C (Table 2). The best correlation was provided by the second-order kinetic model, as was demonstrated by the Langmuir isotherm model. A similar study by Pourfadakari et al. (2017) examined the removal of Cr^6+^ from aqueous solution using nanosized cellulose fibers obtained from rice husk [77] (Table 2). The experiment showed that the adsorption efficiency reached 92.99% at pH = 6, contact time = 100 min, adsorbent dose = 1.5 g/L, and 30 mg/L initial Cr concentration. Additionally, the Langmuir isotherm with (R^2^ = 0.998 at 303 °K) and pseudo-first-order kinetic model (R^2^ = 0.993) were the best models for describing the Cr^6+^ adsorption reactions. In another study, Altun et al. (2016) investigated the biosorption process of Cr^6+^ using rye husk (RH) under various conditions [75]. About 68% of 5.0 mM Cr was removed within 140 min when 0.5 g RH was taken at pH 3 (Table 2). Application of the Langmuir isotherm model yielded maximum biosorption capacity of 0.43 mmol/g at pH 3, where the first-order reversible and pseudo-second-order kinetics models were used to evaluate the data.

Ahmed et al. (2019) used sodium chlorite-modified coir coconut (SCM-CC) for the removal of Cr^6+^ [79]. A total of 99.92% of Cr removal was removed at pH 2 (Table 2). Freundlich isotherm had a better fit than the Langmuir isotherm and the kinetic data were described using Ho’s pseudo-second-order kinetics. Begum et al. (2020) utilized chitosan-coated banana and areca fiber for the removal of Cr^6+^ from wastewater [76] (Table 2). The maximum removal obtained after 150 min at pH 4.5 was 75%. A report on the modification of sawdust for the removal of Cr^6+^ from wastewater was given by Chakraborty et al. (2021) [78]. The maximum removal of Cr^6+^ was found to be 100% at pH 2.0, initial Cr^6+^ concentration of 10 mg/L, and adsorbent dose of 4 g/L. Equilibrium isotherms for the removal of Cr^6+^ were analyzed by the Langmuir, Freundlich, and Temkin isotherm models, and the experimental data were well explained by the Freundlich variant. The maximum adsorption capacity was 8.84 mg/g and the obtained data fitted best to the pseudo-second-order kinetic model.

Shakya and Agarwal (2019) prepared biochar using waste pineapple peel biomass with slow pyrolysis at 350, 450, 550, and 650 °C to explore the effect of temperature treatment on characteristic properties of biochar [147]. Biochar prepared at 350 °C was found to have the highest adsorption capacity of 41.67 mg/g. Complete Cr^6+^ removal was achieved at 10 mg/L Cr^6+^ concentration with all biochars. In a recent study, Saravanan et al. (2021) utilized raw and pyrolysis-assisted dragon fruit peel along with fungal biomass (*Fusarium subglutinans*), a mixed adsorbent, for removing Cr^6+^ from polluted water [148]. Batch adsorption tests reveal that optimum conditions for the effective removal of Cr^6+^ ions onto mixed biomasses (pH = 4.0; biomass dosage = 6.0 g/L for *Fusarium subglutinans*—RDFP, 3.5 g/L for *Fusarium subglutinans*—PADFP; temperature = 30 °C; Cr^6+^ ion concentration = 25 mg/L; equilibrium time = 60 min). The adsorption equilibrium data and contact time data were best fitted to the Langmuir and pseudo-first-order models, respectively. Tytłak et al. (2015) investigated the removal of hexavalent chromium using two potential biochars produced by the thermal decomposition of wheat straw (BCS) and wicker (BCW) [149]. The optimal adsorption capacities were obtained at pH 2 and were 24.6 and 23.6 mg/g for BCS and BCW, respectively. The desorption studies of Cr^6+^ ions in relation to HCl and HNO_3_ concentrations were performed to check the reversibility of biochar. The least amount of desorption was seen for BCW (51%) and the most for BCS (79%), when nitric acid was used as a desorptive agent. For hydrochloric acid, a distinct impact was seen, with BCS desorption being the lowest (39%) and BCW desorption being the highest (47%). This work demonstrated that Cr^6+^ ions do not completely desorb from the surface of biochar, even when concentrated hydrochloric or nitric acid was applied [149].

A comparison between three kinds of adsorbents was performed by Imran et al. (2020) [150]. These authors used novel biochar derived from *Chenopodium quinoa* crop residues (QBC), QBC activated with magnetite nanoparticles (QBC/MNPs), and strong acid HNO_3_ (QBC/Acid) to evaluate their batch and column scale potential to remove Cr (VI) from polluted water. The impact of different process parameters including dose of the adsorbent (1–4 g/L), contact time (0–180 min), initial concentration of Cr (25–200 mg/L) as well as solution pH (2–8) was evaluated on the Cr^6+^ removal from contaminated water. Results revealed that QBC/MNPs proved more effective (73.35–93.62%) for Cr^6+^ removal with 77.35 mg/g adsorption capacity as compared with QBC/Acid (55.85–79.8%) and QBC (48.85–75.28%) when Cr concentration changed from 200 to 25 mg/L.

##### Composites

Composites are used to remove Cr due to easy handling, low cost, high efficiency, improved process ability, surface area, stability, and tunable properties. Different kinds of composites are used for this purpose. Zhou et al. (2016) fabricated a hydrophobic magnetic adsorbent based on polypyrrole coating on acid-dissolved fly ash (MSFA/PPy, which can float on the water body’s surface and easily collected by a magnetic field after adsorption [151]. The biosorption process best fitted the Langmuir isotherm model followed by the pseudo-second-order kinetic model, reaching the highest adsorption of Cr^6+^ which was 66.93−119.33 mg/g at experimental conditions. Islam et al. (2017) prepared bio-composite from cellulose and Bijoypur clay (Kaolinite) exhibited enhanced properties compared to their original counterparts [125]. The best result was found for the composite containing 20% clay and 80% crystalline cellulose at pH 4 and 27 °C which was 2.37 mg/g. Hokkanen et al. (2016) synthesized hydroxyapatite micro-fibrillated cellulose (CHA/MFC) composite for removing Cr^6+^ from wastewater through the batch process, where the maximum adsorption capacity obtained was 2.208 mmol/g at 25 °C [126]. Similarly, Islam et al. (2021) were able to remove 100% of Cr from industrial wastewater using Banana rachis CNC/clay composite in both cases for 40–60% and 30–70% CNC-clay ratio [127]. Rahaman et al. (2021) implemented a biodegradable composite of modified cellulose and chitosan applied to Cr and removed 56% efficiently at pH 4 when the metal concentration and adsorbent dosage were, respectively, 60 mg/L and 1.0 g/L [128] (Table 3).

Mohamed et al. (2017) used the electrospinning method to fabricate polyacrylonitrile (PAN) and carbon nanotube (CNTs)/titanium dioxide nanoparticles (TiO_2_) containing amine groups (TiO_2_-NH_2_) composite nanofibers for the removal of Cr^6+^ from wastewater [191]. The maximum adsorption capacity of PAN-CNT/TiO_2_-NH_2_ for Cr^6+^ was 714.27 mg/g at 293 K at pH 2, and instead of the pseudo-second-order model the process can be better described using the nonlinear pseudo-first-order model. After 5 usage cycles, up to 80% adsorption capacity can be achieved. For the removal of Cr^6+^ anionic species, Huang et al. (2018) used a simple polymer cross-linking method for adsorption in aqueous solutions. Carboxylated multi-walled carbon nanotubes were modified with chitosan to increase adsorption of Cr^6+^ in acidic aqueous solutions (pH = 2), where the maximum adsorption capacities were 143–164 mg/g within only 30 min [192] (Table 3).

On the other hand, Wang et al. (2017) went through a simple hydrothermal method for the preparation of molybdenum disulfide coated Mg/Al layered double hydroxide composites (LDHs@MoS_2_) for adsorption [193]. At pH 5.0, 76.3 mg/g Cr^6+^ was removed relying on ionic strength and pH. Choudhury et al. (2018) fabricated a clay-alumina ceramic composite membrane comprising hydroxyethyl cellulose and CuO nanoparticles for removing Cr^6+^ from contaminated water [129]. Here the maximum percentage of adsorption for Cr^6+^, obtained at 2 bar trans-membrane pressure, was 91.44%. In a more recent report, Dokmaji et al. (2020) modified multiwall carbon nanotubes (MWCNTs) chemically for the removal of both Cr^3+^ and Cr^6+^ from wastewater [130]. Cationic surfactant cetyl trimethyl ammonium bromide (CTAB) was used to modify which resulted in a product named MWCNTs-CTAB. At optimum conditions, it can remove almost 98% of Cr. On the other hand, when MWCNTs are modified using an anionic surfactant, such as sodium lauryl sulfate (SLS) after magnetization with magnetite (M), produces MWCNTs-M-SLS, which can remove 99% of Cr. The isotherm of both components fitted the Langmuir model properly where the adsorption capacities for Cr^3+^ and Cr^6+^ were 66.2 and 27.8 mg/g, respectively. The optimum parameters were: pH = 5, contact time = 60 min (for Cr^3+^) and 30 min (for Cr^6+^), initial concentration = 150 mg/L (for Cr^3+^), and 300 ppm (for Cr^6+^).

Kumar et al. (2020) introduced polyaniline-impregnated nanocellulose (PANINC) composites for the removal of Cr^6+^ from wastewater [194]. Their experiment showed that this composite can remove 92.59 mg/g (96.5%) of Cr^6+^ (Table 3). The optimum contact time and pH were 60 min and 6, respectively, where the initial concentration was observed to be 100 mg/L for the maximum removal of Cr^6+^. The isotherm model fitted the Langmuir model better than the Freundlich model. Wang et al. (2022) used integrated micro-electrolysis composites (IMC) through a facile one-pot method with red mud and rice straw [195]. The maximum adsorption efficiency reached was 97.74% at pH 6. Wu et al. (2022) prepared a polysaccharide-derived composite by embedding carbonized chitosan into triethylenetetramine-modified sodium alginate (CTS/CS-50) [196]. The synthesized composite has exhibited a maximum adsorption capacity of 144.49 mg/g for Cr^6+^ at pH 1.

Stoica-Guzun et al. (2016) used three factorial Box–Behnken Design (BBD) to determine the capacity of bacterial cellulose composites (BC-Fe_3_O_4_) for the adsorption of Cr^6+^ under various conditions [198]. Cr^6+^ can be removed efficiently when the initial pH is 4 with a minimum release of iron. Subedi et al. (2019) used magnetic chitosan (Chi@Fe_3_O_4_) and graphene oxide-modified magnetic chitosan (Chi@Fe_3_O_4_GO) nanocomposite for the removal of Cr^6+^ from water [131]. The maximum adsorption capacities were 142.32 and 100.51 mg/g for Chi@Fe_3_O_4_ and Chi@Fe_3_O_4_GO, respectively. In one study, straw-derived hierarchically porous carbon-supported FeNi bimetallic nanoparticles (FeNi@HPC) were prepared for effective removal of Cr^6+^ from water [132]. Higher temperatures favored the removal of Cr^6+^ and FeNi@HPC manifested the lowest activation energy when compared to Fe@HPC and FeNi NPs. The best conditions for the activity of FeNi@HPC were assessed, and the highest removal efficiency equivalent to 30 mg/L of Cr^6+^ was achieved at pH= 4.0 in 360 min with a dosage of 0.5 g/L.

##### Metal–Organic Framework

Metal–Organic framework (MOF), a porous material, has attracted the attention of researchers because of its tunable structures, porosity, flexibility, and functionality that provide the chances to utilize these materials for multiple functionalities such as catalysis, adsorption, etc. [199]. Generally, two kinds of primary building units are contained by these MOFs, one is the organic linker and the other one is metal core. The organic portion acts as a linker such as terephthalic acid, trimesic acid, fumaric acid, etc. On the other hand, transition metals (Fe, Zn, Cu), p-block elements (In, Ga), lanthanide, and actinide series (La, U, Th) are used for the building of the metal core [200]. A number of researchers have mentioned the utilization of MOFs for the successful removal of different heavy metals from wastewater including Cr. nFe_3_O_4_@MIL-88A(Fe)/APTMS nanocomposite was synthesized using microwave for the removal of Cr^6+^. This MOF was able to remove 7.99 mg/g Cr^6+^ from wastewater when pH was <4 at contact time of 30 min [201]. A water-stable MOF named BUC-17 was synthesized using solvothermal process and was able to adsorb 68.2 mg/g of Cr^6+^ when the adsorbent dosage was 150 mg/L maintained at pH 4 after 500 min [202]. Another study was reported by Han et al. [203] where they synthesized La-Zr bimetallic MOFs containing the molar ratio at 1:1 (1LaUN_12_) for the efficient removal of Cr^6+^ from wastewater. Maximum adsorption of Cr^6+^ was 222.5 mg/g at pH 2. Moreover, above 40% of Cr^6+^ was also reduced to less toxic Cr^3+^ by amino groups and immobilized over the surface of 1LaUN_12_ [203]. Although MOFs have promising properties and are good at removing Cr, they have poor chemical stability. In order to implement MOFs into industrial wastewater applications, further research is required to optimize their structures and scale them up. Further, different functionalization methods must be proposed and applied to enhance MOF stability and sorption kinetics.

##### Mesoporous Silica

Because of its exceptional surface features, which include thermal/chemical stability, high specific area, low casting, low density, and a range of pore volume and distribution, porous silica has been used for a long time as inorganic catalysts and supporting materials [204]. Porous silica’s morphology, pore size distribution, and structural characteristics can be easily modified as compared to those of other porous materials, such as carbonaceous and zeolite, to allow for greater flexibility in meeting the demands of various applications [205]. On the other hand, the pore size of porous silica has demonstrated the impact of pore structure on products and is connected to selectivity and catalytic activity. Because of its spongy architecture and high thermal/chemical stability, researchers have spent the last two decades concentrating on the meso-class of porous silica with nanoparticle size [205,206,207]. The flexibility and great selectivity of the internal and exterior surface of mesoporous silicates, which are their best qualities, can generally be altered with a variety of inorganic/organic functional groups [208,209,210]. Tetraethyl orthosilicate (TEOS), an alkoxysilane precursor, is typically hydrolyzed to create mesoporous silicas, which then condense and polymerize in the presence of a suitable catalyst and template to produce a network of siloxanes (-Si-O-Si- links) (surfactant) [210]. Mesoporous silicas are widely employed with surface modification for the adsorption of heavy metals because they have outstanding characteristics [211].

By grafting N-(3-trimethoxysilylpropyl) diethylenetriamine (DAEAPTS) onto SBA-15, Kim et al. (2018) produced two forms (powdered and granular) of mesoporous silica for the removal of Cr^6+^ [212]. Batch experiments revealed that the Cr^6+^ sorption was favorable at acidic pH conditions with the greatest sorption at pH 3 reaching the equilibrium within 10 min and the maximum sorption obtained was 330.88 mg/g [212]. The chromisorption of chromium also followed a pseudo second-order kinetics in mesoporous silica magnetic nanoparticles modified with 3-aminopropyl-triethoxyxilane. The Langmuir adsorption isotherm was able to fit the adsorption data with high accuracy, and the highest adsorption capacity was 185.2 mg/g [213]. For the purpose of chromium adsorption, mesoporous silica surfaces functionalized with monoamino [3-aminopropyl trimethoxysilane] (APTMS) and triamino [N-(3-trimethoxysilyl propyl) diethylenetriamine] (DETA) were employed. It was discovered that APTMS-MCM-41 displayed greater adsorption capacity than DETA-MCM-41. The maximum adsorption reported was 111.1 mg/g at pH 3 [214]. Hexavalent chromium is completely removed by a composite of microporous silica and hierarchical hollow multi-shells. The material demonstrated an adsorption capacity of 257.57 mg/g at 20 °C at an optimal pH of 4 with a loading of 5 mg because of its large surface area and extremely small 1.22 nm pore size [215,216].

##### Zeolites

Zeolites are aluminosilicate minerals with micropores that have various cavity structures made of a three-dimensional framework and a negatively charged lattice. Na^+^, K^+^, Ca^2+^, Mg^2+^, and other cations that can readily exchange with other cations in the solutions balance the negative charge. Zeolites are appealing adsorbents for the removal of heavy metal ions from aqueous systems due to their high specific surface areas, strong ion-exchange capacity, and comparatively inexpensive cost [217]. The use of synthetic zeolites for heavy metal sequestration in wastewater treatment has been found to have the greatest applicability, followed by modified zeolites. Due to the fact that natural zeolites’ mineralogical composition varies widely between regions and even within a single mineral deposit, natural variants, despite being extremely desirable from an economic standpoint, show the lowest metal sorption for the majority of heavy metals [218]. In addition, numerous additional minerals that behave as pollutants due to their low metal sorption capacity are frequently found in the ore along with other varieties of zeolite. Quartz, albite, biotite, illite, montmorillonite, feldspar, calcite, halite, and heulandite are among the contaminating minerals that are frequently found [219,220]. Adsorbents with remarkable uniformity in their characteristics, such as pore size distribution, hydrophobicity/hydrophilicity, and the presence of a single compensatory cation, are synthetic zeolites, which are typically composed of a single phase. These factors taken together ensure that they have a higher cation exchange capacity than natural zeolites [221].

The removal of Cr^6+^ was possible after modification of clinoptilolite using hexadecyl pyridinium bromide which was not achievable in case of natural or synthetic zeolites [222]. Neolaka et al. (2022) utilized activated natural zeolite-magnetic composite (ANZ–Fe_3_O_4_) adsorbent material for the removal of Cr^6+^ from synthetic wastewater [223]. The outcome showed that the best adsorption took place at a pH of 2, with an adsorbent mass of 0.20 g, for 50 min at a temperature of 298 K [223]. For the purpose of removing Cr^6+^ from wastewater, synthetic zeolite spheres filled with nanoscale Fe-Al bimetallic oxide were produced. With an initial Cr^6+^ concentration of 20 mg/L (pH = 3), the results showed that nano Fe-Al bimetallic oxide was an efficient material for removing Cr^6+^, with a maximum removal efficiency of 84.9% [224]. Clinoptilolite, a natural zeolite, was used to create hollow fiber ceramic membranes (HFCM) to study the adsorptive removal of hexavalent chromium, Cr^6+^, from an aqueous solution. At a pH of 4 and a Cr^6+^ concentration of 40 mg/L, the HFCM performed in adsorption/filtration with a 44% removal of Cr^6+^ efficiency [57].

Due to simple operation, broad applicability, high removal rate, and affordable reusability, adsorption has become the most promising and widely investigated technology for removing chromium from wastewater. However, the present methods of surface modification require intense heat and pressure, powerful acids and bases, or vigorous oxidation and reduction reactions. The carbon-based adsorbents are expensive due to this labor-intensive preparation process, which limits their widespread use in industrial applications. On the other hand, chitosan-based adsorbents exhibit limited reusability without adjustments. Strong bonds (between the metal ions and the adsorbent surface) may be responsible for this behavior, as well as low thermal/chemical stability, low mechanical strength, incomplete desorption, a decline in the efficiency of the adsorbate–adsorbent interaction, and a lack of adsorption sites. The dosage of biosorbent has a significant impact on the removal effectiveness because it provides more active biosorption sites. At higher temperatures, bond rupturing, Gibb’s free energy reduction, and decreased solution viscosity may all contribute to an increase in the biosorbent capacity. These factors boost the biosorbent active sites and raise the collision frequency (mobility and kinetic energy) between them and metal ions, which results in a higher affinity [225]. At higher temperatures, the bonding force between the biosorbent and the contaminants might weaken, which would result in less biosorbent being absorbed. It was revealed that as the mixed agitation rate increases, the elimination efficiency rises [226]. In case of MOF though it has good capability to remove Cr efficiently, sometimes it contains micropores that are inaccessible for the target material and most of them are highly unstable in water. MOFs have been fabricated using Mn, Fe, and Cu, although the majority of them have poor chemical stability. In order to scale up these materials and fine-tune the MOFs’ structure for use in industrial wastewater applications, more study is still required. Moreover, various functionalization techniques should be suggested and used to improve the sorption kinetics and stability of MOFs. So, it is still a challenge for researchers to develop an adsorbent with the qualities such as low-cost materials, high uptake, and efficient regeneration processes.

### 2.2. Electrochemical Treatment

Electrochemical treatments of wastewater have not received much consideration in view of the need for enormous capital cost and power supply, which is also expensive [173,227]. However, in accordance with stringent ecological guidelines for wastewater exposure, electrochemical technologies have become very significant worldwide in the last two decades [228]. Electrochemical Cr^6+^ decrease strategies can be utilized, contingent upon the pH of the fluid arrangement, the power of the current density, and the electrode material utilized [229]. In an electrochemical system, oxidation reaction occurred in the anode (positive side) and the reduction process occurs at cathode (negative side), where the electrons transfer. These two chemical reactions are called redox (reduction–oxidation), leading to Cr removal from wastewater. Selection of the anode and cathode mainly decides the type of the electrochemical method and influences the removal efficiency towards specific metal ions [230].

A number of simultaneous procedures could occur on the outer layer of the electrode or in the given solution [231]. In this review, some significant electrochemical treatment technologies for the removal of Cr including electrocoagulation (EC), electro flotation (EF), electrochemical reduction (ER), and electrically driven ion transport are described.

#### 2.2.1. Electrocoagulation

Electrocoagulation (EC) is a straightforward and gainful innovation utilized in wastewater treatment industries [232]. However, it was never deemed to be a dependable technique because of its poor efficient reactor structure and issues of electrode dependability [227]. As of late, EC is known as a little-scale wastewater treatment strategy with improved specialized techniques. Removal of Cr by EC is tabulated in Table 4.

##### Iron Electrodes

A number of parameters, for example, pH, applied electrical current, and application time can affect the efficiency of Cr^6+^ removal using iron electrodes and a high efficiency (>90%) can be obtained at optimum conditions. There are two stages involved in the electrocoagulation system. Firstly, Cr^6+^ reduces to Cr^3+^ either directly at the cathode or by Fe^2+^ ions obtained from the oxidation of iron anode, and secondly, co-precipitation of the Fe^3+^/Cr^3+^ hydroxides is formed subsequently. The reduction of Cr^6+^ to Cr^3+^ by Fe^2+^ ions is favored at low pH, while conversely, the precipitation of Fe^3+^/Cr^3+^ hydroxides occurred at high pH (>3) because metal hydroxide species (both chromic and iron hydroxides) are soluble at low pH [245]. Kim et al. (2020) examined the removal of Cr from wastewater using iron electrode. They found that the optimum pH of this process was 7–9 while the mass of sludge formed and the amount of energy consumed were 0.68–2.5 kg/m^3^ and 0.37–2.78 kW h/m^3^, respectively [246]. The process fitted both the first-order and second-order kinetic models. Lu et al. (2016) successfully removed Cr^6+^ from aqueous solution using iron electrode via the EC process. The optimum conditions were: pH = 2; current density = 0.73 mA/cm^2^; electrolysis time = 50 min [242]. Verma et al. (2013) were able to remove 100% Cr for both Cr^3+^ and Cr^6+^ and hexavalent Cr, for an electrolysis time lasting 45 min at 4 pH with current density of 50 mA cm^−2^ [241].

##### Aluminum Electrodes

Al (OH)_3_ acts both like an adsorbent and trap to separate heavy metals [247]. An investigation directed by Mansoorian et al. (2014) demonstrated that larger amounts of metal ions can be removed by the electrocoagulation process using adsorbents such as hydroxide iron and steel flocs [240]. Mahmad et al. (2016) utilized aluminum electrodes for the removal of total Cr and 72.65% of total Cr was removed at pH 3 and the voltage was 2.5 V [248]. Elabbas et al. (2016) removed 99% Cr using Al electrode when the current density was 400 A/m^2^ [243]. The pH was 5.3 and it took 360 min for the process to be completed. Recently, Lu et al. (2022) investigated the EC technique with Fe and Al anode for the elimination of Cr^6+^ from wastewater at initial pH of 3.5–4.0 [244]. The study showed that the EC with Al-Al-Al-Al combination (anode–cathode-anode–cathode) exhibited the highest removal efficiency among different electrode combination modes, whereas the EC with Fe-Fe-Fe-Fe combination exhibited the poorest removal efficiency. At the initial pH of 3–6, Al EC displayed desirable removal efficiency for Cr^6+^ (84.2–96.4%), and total Cr (83.1–94.9%). At the conductivity of 899–2300 μS/cm, excellent pollutant removal efficiency was maintained (90.2–99.8%).

#### 2.2.2. Electro-Floatation

Today, heavy metal contaminants can be removed using the electro-flotation (EF) process [249,250,251]. since different strategies for treating wastewater for the most part do not work productively for extremely weakened arrangements (fixations underneath 50 m/gm^3^) [252]. EF became highly regarded in light of its flexibility, straightforwardness in plan and activity, ecological sensitivity, low operational expenses, and little and reduced units [253]. To overcome the impediments of the procedures referred to previously, a few examinations consolidated EC and EF, and it prompted higher expulsion effectiveness in contrast to utilizing only one. This joint strategy is an increasingly advocated and successful approach to expel toxins [254]. The blending of EC and EF is called electrocoagulation-floatation (ECF) and the strategy leads to better expulsion rates. Various analyses were undertaken to explore the impacts of the working conditions on the expulsion of substantial metals. However, the completion of this process for the removal of Cr is rare. Zouboulis et al. (2003) removed Cr using a process where 95% of Cr was removed successfully [255].

#### 2.2.3. Electrochemical Reduction

As the sacrificial anodes are consumed during the electrocoagulation process, they should be replaced occasionally. There are alternative options for the electrochemical removal of Cr^6+^ using non-sacrificial cathodes, which are: (i) Cr^6+^ is directly or indirectly reduced to Cr^3+^, and (ii) using the adsorption or precipitation process [256]. The electrode materials and reactor arrangement study the removal of Cr^6+^ to a greater extent. It is noted that lead dioxide (PbO_2_) coatings and noble metal oxides (dimensionally stable anodes, DSA) coatings on titanium (Ti) are generally used as the non-sacrificial anode electrode for commercial purposes due to the oxidative and destructive properties of Cr^6+^ [257]. The productivity and procedure cost execution at regular cathode materials, for example, Fe and Cu, is not that fulfilled [258]. Subsequently, significant endeavors have been conducted to generate better cathode materials and increasingly proficient expulsion procedures for electrochemical Cr^6+^ reduction.

##### Carbon-Based Electrode

Wang and Na (2014) detailed the Cr^6+^ evacuation utilizing carbon nanotube (CNT), and it exhibited legitimately developed on stainless steel mesh (SSM) [259]. At first, the reduction of Cr^6+^ was completed by utilizing negatively polarized electrode surface area and then the same electrode attracted the adsorbed Cr^3+^ cations, namely Cr^3+^ and Cr(OH)^2+^ by an electrostatic force. In this way, the anode can be recovered by turning around the polarization to discharge Cr^3+^. The electrochemical Cr^6+^ reduction performance is determined by various parameters in the corresponding regions [260].

##### Gold Electrodes

A number of experiments have been conducted by Jin and Yan (2015) for the electrochemical reduction of Cr^6+^-consuming gold-based electrodes. These authors confirmed that it, however, shows greater activity in alkaline solution [261]. As a result, for the electrochemical reduction of trace amount of Cr^6+^, Jin et al. (2014) used gold nanoparticle-enriched TiO_2_ nanotube clusters [262]. The electrodes were arranged with metal–semiconductor heterojunction infrastructure and large outer area in an exceptional way, which boosted its activity 23 times greater than that of polycrystalline gold electrodes. It resulted in a potential lignin-treated adsorption–discharge method, which enhanced the electrochemical evacuation of Cr^6+^. Two levels of pH were used: 2 and 11. The former pH was maintained for the adsorption and initial concentration of Cr^6+^, while the latter was used for the discharge and electrochemical decrease. Insoluble Cr(OH)_3_ can be obtained from discharged Cr^6^ which was then effectively isolated.

##### Conducting Polymers

To commercially reduce Cr^6+^ efficiently from wastewater, an unconstrained electron donor, conducting polymer, was successfully tested in 1993 [263]. In order to provide a bulk surface area, conducting polymer films are accumulated on substrates followed by submerging in Cr^6+^ solution. The application of polypyrrole (PPy) films onto an aluminum substrate, the electro-polymerization adherent, for the reduction of Cr^6+^ was narrated by Conroy and Breslin (2004) [264]. If the subsequent electrodes are reduced to a negative potential without Cr^6+^, the consumed catalyst can be regained. The polypyrrole (PPy)- modified electrode is much more effective for the same purpose because it is more effective than stainless steel (SS) [265]. Under stirring conditions, 92% of Cr^6+^ was removed (PPy electrode), 18% (SS electrode) in the potentiostatic (PS) process, 100% (ppy electrode), and 36% (SS electrode) in the potentiodynamic (PD) process, respectively. The PPy stability under PD was not as effective as PS conditions. Because of quick reaction and greater stability, polyaniline (PANI) is considered to be a reasonable material according to Ruotola et al. (2003) [266]. Almost 100% Cr^6+^ can be reduced PANI film and no significant degradation occurs in polyaniline due to the cathodic protection technique. The reaction rates of Cr^6+^ reduction observed at reticulated vitreous carbon (RVC) and PANI electrode were much higher than that of the uncovered RVC electrodes when they were compared [267,268]. Graphene–polyaniline (GR-PANI) electrodes were blended through a combination of electrochemical polymerization and chemical system by Gao et al. (2011) [269]. The GR-PANI showed enormous sensitivity and an outstanding electrocatalytic action in reducing Cr^6+^ due to its bulk surface area and unfastened structure.

#### 2.2.4. Electrically-Driven Ion Transport

To reduce energy consumption and waste production, the membrane technology integrated and optimized with the electrochemical strategy has been developed to treat Cr^6+^ from wastewater. Without affecting the physical condition or consuming chemical substances, synchronous concentration and separation are achieved and the elementary is the recommended position of a membrane procedure. This process is specifically implemented for the removal of Cr^6+^ electrochemically [270].

##### Electrodialysis and Electro-Electrodialysis

Electrodialysis is used in wastewater treatment, desalination, and electrolyte detachment. When an electrodialysis system is used for the treatment of wastewater, an anionic layer hinders the movement of the other metallic ion pollutants while allowing Cr^6+^ to move towards the anode through an anionic layer [271]. Due to the increasing ion transport at lower pH, efficient removal of Cr^6+^ at a lower concentration is possible [272]. Chen et al. (2009) utilized monovalent-specific electrodialysis layer in order to build up an electrodialysis system to remove Cr^6+^ when the electroplating wastewater was at low pH [273]. When the current density and stream rate were high along with greater membrane area, chromate recovery was enhanced.

##### Electro-Deionization

Ion exchange resin, a solid conductive ion medium has been introduced into the dilute compartment to overcome drawbacks. That is how a highly efficient ion separation and energetically proficient technology has been developed by joining two different methods, ED and ion exchange process. The synergistic arrangement of these two technologies is called electro-deionization (EDI) or continuous electro-deionization (CEDI). In this method, ion exchange resins are used as a conductor between the ion exchange films. Moreover, by splitting water into H^+^ and OH^−^ ion exchange resins can be recovered to their proton and hydroxide forms. A strong potential large-scale reticular anion exchange resin (Amberlite^®^ IRA900) with a high limit with respect to Cr^6+^ transport (116 mg Cr^6+^ per gram of resin) was introduced by Alvarado et al. (2009) [274]. Consuming this anionic resin and Amberlite^®^ 200C, about 98.5% removal of Cr^6+^ became possible under 0.07 kWh/m^3^ energy consumption in a continuous electro-deionization process. A fraction of the developed H^+^ and OH^−^ ions recombine before recovering the resins at monopolar film and by using EDI with bipolar layers, additional improvement can be obtained in treatment proficiency [275]. Though ED or EDI-based partitions need more refinement they are currently an efficient technique for clean evacuation of Cr^6+^ [275,276].

Due to electrode passivation and significant electrical energy consumption, the electrochemical process is a highly expensive technological procedure. To improve the effectiveness of the wastewater treatment, more consideration should be given to the reactor design and operating conditions in case of ER. The issue of energy consumption needs to be resolved in order to commercialize this kind of treatment for use in industrial applications. Electrode passivation and relatively high energy consumption are EC limitations, along with the difficulty of large-scale applications at lower energy consumption [277,278]. Several effective methods, including aggressive ion-addition, alternating current operation, polarity reversal operation, ultrasonication, mechanical cleaning of electrodes, chemical cleaning of electrodes, and hydrodynamic scouring, have been suggested to reduce the passivation of the electrode. Each option, however, has disadvantages, including the development of hazardous byproducts, high costs for extra infrastructure and treatment, and increased sludge production [279]. As a result, the EC process is still not entirely applications mature.

### 2.3. Physico-Chemical Processes

Physico-chemical treatment of wastewater focuses primarily on the separation of colloidal particles. This is achieved by adding chemicals that modify the contaminants’ physical state. This process may consecutively be performed in a single unit or in separate units. This process includes the ion exchange method, chemical reduction, photocatalytic reduction, etc.

#### 2.3.1. Ion Exchange Method

Scholars have studied ion exchange methods extensively and they have been viewed as a suitable process for the removal of Cr at low concentrations [43,44,280]. The ion exchange method is a reversible chemical reaction used to replace undesirable metal ions with harmless and environmentally friendly ones. Cr is removed by attaching it to an immobile solid particle as a replacement for the solid particle cation. The material of solid ion-exchange particles could be either natural, e.g., inorganic zeolites, or synthetically produced, e.g., organic resins [230]. Cr^3+^ and Cr^6+^ can effectively be removed using a two-step ion exchanger, i.e., cation and anion resins, respectively. Strong basic anion exchangers containing an exchangeable counter ion of Cl_2_ are commonly used for the removal of Cr^6+^ [44]. There are a number of ion exchange resins such as strong anionic resins (e.g., Amberlite IR and IRA-900, DOWEX 1), weak anionic resins (e.g., Amberlite IR 67RF and IRA-94, DOWEX MA43, and MAC3) and cation exchange resins (e.g., Amberlite IR-120, IRN77, and SKN1) [43,44]. Effluent water passes through Amberlite IR-120 and Amberlite IRA-402, which removes Cr^6+^ [37].

Generally, weak base anion exchange resins are used to remove chromates from water under acidic pH values. The resins keep a stoichiometric ratio with sodium hydroxide when regenerated. On the other hand, strong base anion exchange resins can only be used when it is necessary to remove trace amounts of chromate from tap water. Using concentrated NaCl (5–8%) these resins can be regenerated which can subsequently be improved by adding NaOH to the solution to transform the resin from HCrO_4_^−^ to CrO_4_^2−^. However, the need for continuous regeneration and concentrate disposal, dynamic fouling of the resins, and the effect of other ions present in the water are the disadvantages of the discussed method.

Using an ion exchange process, Tiravanti et al. (1997) conducted the pretreatment of tannery wastewater for the removal and recovery of Cr^3+^ [281]. They found that, compared to the traditional treatment, the process requires minimal costs for sludge treatment and disposal since sludge production fell by 80% and showed a significant reduction of chemical compounds. El-Moselhy and Hakami (2015) utilized carminic acid-modified anion exchanger (IRA 900) for the removal of Cr^6+^ [282]. Maximum adsorption occurred when the pH was between 4.0 and 4.5 and the adsorption isotherm fitted the Langmuir model best. The adsorption capacity was 19.27 mg Cr^6+^/g of the adsorbent. Zang et al. (2018) conducted an experiment for the removal of Cr^6+^ using Poly-epichlorohydrin-dimethylamine (EPIDMA) modified weak base anion exchange resin D301 [283]. The maximum adsorption capacity was 194 mg/g at 25 °C and at pH 2. The kinetic data were best fitted by the pseudo-second-order kinetic model while the batch equilibrium data followed the Langmuir isotherm model well.

#### 2.3.2. Reduction Process

Reduction reactions chemically convert hazardous pollutants into less toxic compounds that are less mobile or inert. In this process, Cr^6+^ adopts an electron and converts it into Cr^3+^. According to the process of electron generation, the reduction process can be classified into chemical and photocatalytic reduction. These are explained in more detail below.

##### Chemical Reduction

In the case of chemical reduction process, the electron is generated from a chemical reaction. A reducing compound is used to reduce Cr^6+^ to Cr^3+^, for example, sulfur compounds, iron salts, etc.

1. Sulfur Compounds

The formation of Cr^3+^ hydroxide precipitation from Cr^6+^ is the widely accepted typical procedure for treating chromate-containing rinse water. Commonly, industries use acidic solutions of sulfur dioxide gas or sodium bisulfite as a reducing agent. In order to neutralize the acidity, NaOH solution or Ca(OH)_2_ slurry is used to treat the effluent and precipitate the Cr. It has been advised to use NaOH when the sludge formation needs to be minimized as much as possible [284]. The formation of a large amount of residual sludge, which creates difficulties in managing, transporting, final disposal issues, and associated cost, is one of the major problems of these techniques. When the initial concentration of Cr^3+^ was 100 mg/L, the percentage removed reached 99.37% to 99.6% at pH 7–11 [273].

2. Iron Salts

Iron can reduce Cr^6+^ generally under acidic conditions. Hexavalent Cr is reduced to trivalent state using FeCl_2_ and FeSO_4_ at low pH, given that Fe (II) and (III) appears as free ions, for subsequent precipitation [285].

6Fe^2+^ (aq) + Cr_2_O_7_^−2^ (aq) + 14H^+^ (aq) → 6Fe^3+^ (aq) + 2Cr^3+^ (aq) + 7H_2_O

Cr^3+^ (aq) + OH^−^ (aq) → Cr(OH)_3_(s)

Fe (II) requires highly acidic conditions to remain in the solution, despite the fact it remains a free ion up to a pH of 4.7. Better reduction rate can be achieved at low pH due to charge distribution and spatial configuration changes. One study shows that the reduction kinetics of Cr^6+^ remain slow at pH 3.7 but stay stable for months and even years. To enhance the reduction reaction the pH must be less than 3 [285].

Using FeSO_4_ or Na_2_SO_3_ as a reducing agent, Cr^6+^ is chemically reduced to its trivalent state followed by the formation of precipitation with alkali. This process has some problems. For example, using FeSO_4_ will produce solid Fe(OH)_3_ as waste which should be disposed of immediately. On the other hand, toxic SO_2_ is produced, causing air pollution when Na_2_SO_3_ is consumed. Moreover, none of them is suitable for treating dilute Cr^6+^ solution [285].

##### Photocatalytic Reduction

1. Organic Matter for Cr^6+^ Reduction

Under predominant acidic conditions, some dissolved organic matter (DOM) shows very slow but appreciable Cr^6+^ reduction kinetics. Metal reductants such as zerovalent iron, aqueous Fe (II), Fe (II) hydroxides, adsorbed Fe (II), and Fe (II)-chelates, perform better in comparison with organic reductants [286]. The rate of Cr^6+^ reduction can be enhanced due to the complexation of Fe (II) and DOM when Fe (III) is released as a humic acid solution. It is hypothesized that there is some unknown reductant present in humic acid, which is mainly responsible for the reduction of Fe (III) to Fe (II). It is very clear that Fe (II), which is oxidized during the reduction of Cr^6+^, can be recycled. As a result, Fe(OH)_2_ is a stronger reductant than Fe (II)–DOM complexes and is formed by redox-active fulvic acid moieties depending on pH during this process [286]. Humic acid (HA), when coupled with Fe nanoparticles occupies the reactive sites of the surface and curtails the reduction of Cr^6+^, creating both synergistic and antagonistic outcomes.

Conversely, the reduction of Cr^6+^ can be enhanced due to the presence of quinone compounds in HA which acts like an electron shuttle. Agglomeration can be prevented and inhibitory effects can be counteracted by using HA; thus, the nanoparticles are stabilized [287]. The remediation rate of the Cr^6+^-Fe (0) complex can be increased and protection can be provided on the surface of iron by adding benign naturally occurring organic molecules including α-hydroxyl carbonyl, α-hydroxyl carboxylate, α-carbonyl carboxylate, phenolate, carboxylates and/or thiol groups, siderophore, ascorbic acid, or chelating agents such as ethylenediaminetetraacetic acid derivates and acetylacetone [288]. Wu et al. (2022) prepared a polysaccharide-derived composite by embedding carbonized chitosan into triethylenetetramine-modified sodium alginate (CTS/CS-50). The amount of Cr(VI) removed could reach 98% within 80 min under UV light irradiation [196].

2. Fe (III) Photocatalytic Reduction of Cr^6+^ by Organic Acids

Research has focused on Cr^6+^ reduction by organic acids using the photocatalytic impact of Fe (III). The key stage of this reduction reaction is the formation of Fe (III)-organic acid complex which is significantly feasible when applied to organic acids containing an α-OH group [289].

3. TiO_2_ Photocatalytic Reduction of Cr^6+^ by Organic Acids

At pH 3, the photocatalytic reduction capability of TiO_2_ has been studied for the removal of Cr^6+^ due to its properties of oxidizing organic compounds and reducing the metal ions simultaneously. Li et al. (2016) conducted an experiment to remove Cr^6+^ using a TiO_2_-graphene hydrogel with a three-dimensional (3D) network structure facilitated by the photocatalytic reduction process. A total of 100% Cr^6+^ was removed from the solution containing 5 mg/L within 30 min under UV irradiation [290]. Naimi-Joubani et al. (2015) removed Cr^6+^ using illuminated ZnO/TiO_2_ composite [291]. The efficiency in removing Cr^6+^ was 99.99% when the UV/ZnO/TiO_2_ process was used. The photocatalyst dosage was kept at 4 g/L at pH 3 and the process suited the Langmuir–Hinshelwood (L–H) model well. De Bittencourt et al. (2020) used bio-photocatalysts which were successfully prepared by direct coating of the raw and protonated (immersion into 0.2 M HNO_3_) brown algae *Laminaria hyperborea* with FeCl_3_, AlCl_3_, and TiO_2_ solutions [292]. The results showed that the material previously protonated and coated with iron presented the best Cr^6+^ removal results, reaching an efficiency of 100% after 90 min.

4. Photocatalytic Reduction of Cr^6+^ by Ag/Ag_3_PO_4_/Reduced Graphene Oxide Microspheres

The photocatalytic process is considered a simple process for the removal of Cr that uses light and semiconductors. Three key steps are taken in this process: charged carrier photogeneration, charged carrier separation and diffusion to the photocatalyst surface, and redox reaction on the photocatalyst surface [293]. Despite having many positive advantages such as in-site generation of reactive radicals, no chemicals used, and no sludge production; this technology has some drawbacks. It is still on a laboratory scale, low throughput, dependent on pH, and inefficient when different metals are present [294]. Liu et al. (2020) conducted an experiment for the removal of Cr^6+^ from wastewater using Ag/Ag_3_PO_4_/reduced graphene oxide microspheres [295]. With controlled ratios, Ag/Ag_3_PO_4_ components were well distributed on reduced graphene oxide (rGO) sheets using polydopamine as a good reductant and effective linker. More than 90% of Cr^6+^ removal became possible under a continuous photocatalytic system for more than 30 h.

Moreover, Li et al. (2017) achieved rapid Cr^6+^ reduction using Mn_3_O_4_@ZnO under simulated sunlight irradiation at 95.3% in 110 min [296]. Du et al. (2019) used a metal–organic framework (MOF) and UiO-66-NH_2_(Zr/Hf) membrane as photocatalysts to reduce Cr^6+^ ions. They successfully removed more than 94% of it after 20 cycles [297]. Qi et al. (2020) prepared a number of composites using ZnS-Ga_2_S_3_, a flower-like hierarchical heterojunction, which has photo-catalytical properties [298]. Among the composites, ZnS-Ga_2_S_3_-3(ZnS/Ga_2_S_3_ molar ratio 3:1) proved to be the best. The adsorption capacity of ZnS-Ga_2_S_3_-3 was 54.42 mg/g, and its total removal efficiency was 99.10% for 100 mg/L Cr^6+^ solution after 160 min. The Langmuir, pseudo-second-order, and first-order models well described the adsorption isotherm, adsorption kinetics, and photo-reduction kinetics of ZnS-Ga_2_S_3_, respectively. Zhou et al. (2022) prepared nZVI/ZIF-8 MOF nanocomposites for the effective removal of Cr^6+^ [197]. More than 99% was removed from wastewater using nZVI/ZIF-8 nanocomposites at pH 5. Photocatalytic degradation and reduction of available used catalysts in the literature is described in Table 5.

Photocatalytic reduction technology has significant limitations despite the fact that it produces reactive radicals on-site, without the need for chemicals or the creation of sludge. It is still laboratory-scale, has a low throughput, is pH-dependent, and is ineffective when there are different metals present [294]. In the case of the ion exchange technique, further study is needed on the stability and reusability.

### 2.4. Biological Removal

Biological removal of Cr^6+^ refers to microbial removal. The process consists of three stages: firstly, Cr is bound to the cell surface; secondly, Cr is translocated into the cell; and thirdly, Cr^6+^ is reduced to Cr^3+^ [311].

#### 2.4.1. Aerobic Cr^6+^ Reducing Bacteria

Cr^6+^ is first reduced to the short-lived intermediates Cr^5+^ and/or Cr^4+^ and then finally reduced to the thermodynamically stable end-product Cr^3+^ when bacterial reduction of Cr^6+^ takes place in the presence of oxygen. Cr^6+^ is regenerated when Cr^6+^ goes through a one-electron redox cycle by transferring the electron to oxygen. The process generates a reactive oxygen species (ROS) that easily combines with DNA–protein complexes. However, it is quite unclear if the reduction reactions are spontaneous or enzyme-mediated [312].

Cr^6+^ can be reduced using aerobic heterotrophic cells, non-growing cells, growing cells with chromate reductase activity, and growing cells that have lost chromate reductase activity. When *Bacillus* sp. and *Staphylococcus capitis* are employed, the influence of proteins and electron donors such as glucose, fructose, sucrose, and bagasse extract enhanced the reduction rate of Cr^6+^ [313]. Among them, glucose is the best electron donor [314]. Cr^6+^ can be reduced by *Brevibacterium casei* with an azo dye Acid Orange 7 (AO7) and this is a new mechanism. AO7 was used as an e^−^ donor by the reduction enzyme(s) of *B. casei* under nutrient-limiting conditions. The oxidized AO7 produces complex with the resultant Cr^3+^ to form a purple-colored intermediate [315]. Yu et al. (2016) tested *Pseudomonas brassicacearum* LZ-4 for the removal of Cr^6+^ from wastewater and showed that this immobilized strain could remove 80% of 10 mg/L Cr^6+^ [316]. A total of 93% Cr^6+^ (10 mg/L) was successfully removed by An et al. (2020) using *Pseudomonas aeruginosa* strain G12 in wastewater [317].

#### 2.4.2. Anaerobic Cr^6+^ Reducing Bacteria

The reducing activities for Cr^6+^ differ from aerobes in the case of anaerobes where the process is associated with their electron transfer systems. These catalyze both the electron shuttle and respiratory chain [312]. Cr^6+^ can be reduced using micro-organisms under anaerobic conditions with glucose as described below [311]:

CrO_4_ ^2−^ (aq) + 8H^+^ (aq) + 3e^−^→ Cr^3+^ (aq) + 4H_2_O

Cr^3+^ (aq) + 4H_2_O → Cr(OH)_3_(s) + 3H^+^ (aq) + H_2_O

C_6_H_12_O_6_ + 8CrO_4_ ^2−^ (aq) + 14H_2_O → 8Cr(OH)_3_(s) +10OH^−^ (aq) + 6HCO^−^ (aq)

The anaerobic reduction potential of *Pannonibacter phragmitetus* is better than the aerobic variant under alkaline conditions, showing a potential application for Cr^6+^ detoxification [318]. *P. phragmitetus* cells coated with polyethylenimine-functionalized magnetic nanoparticles can reduce Cr^6+^ efficiently and be easily separated from reaction mixtures by magnetic force. The results of the magnetically immobilized cells prove that the magnetic cell separation technology can be efficient when applied to Cr^6+^ removal from alkaline wastewater [319].

The rapid anaerobic removal and reduction of chromate by the rest of *E. coli* cells were significantly enhanced by: firstly, the quinone redox mediators lawsone, menadione, anthraquinone-2-sulfonate, and anthraquinone-2,6-disulfonate; and secondly, the addition of glucose as an electron donor [320]. Cr^6+^ was adsorbed by chitosan beads and bio-reduced by *E. coli* cells which made possible the bio-regeneration of the chitosan beads after *E. coli* biofilm had grown significantly [321]. Moreover, chromate reduction was enhanced by these cells in the presence of quinone redox mediators [322]. Biogenic iron (II) and sulfides generated by IRB (iron-reducing bacteria) and SRB (sulfate-reducing bacteria) show 100 times faster reduction than CRB alone. H_2_S, a Cr^6+^ reductant, is produced from SRB and conducts the process through three stages [323]: (i) sulfate reduction, (ii) chromate reduction by sulfides, and (iii) precipitation of Cr^6+^ by sulfide:

SO_4_^2−^ (aq) + 2CH_2_O + H^+^ (aq)→ HS^−^ (aq) + 2H_2_O + 2CO_2_(g)

8CrO_4_ ^2−^ (aq) + 3HS^−^ (aq) + 17H_2_O → 8Cr(OH)_3_(s) +3SO_4_ ^2−^ (aq) + 13OH^−^ (aq)

Cr^6+^ (aq) + 3HS^−^ (aq)→ CrS_3_(s) +3H^+^ (aq)

Fe (II), produced from Fe (III) using IRB, reduces the hexavalent Cr into trivalent [323].

C_6_H_12_O_6_ + 24Fe^3+^ + 12H_2_O → 6HCO_3_^−^ + 24Fe^2+^ + 3OH^−^

3/4C_3_H_5_O_3_ ^−^ + 3Fe(OH)_3_(s)→ 3/4C_2_H_3_O_2_ ^−^ + 3Fe^2+^ + 3/4HCO_3_^−^ + 2H_2_O + 1/4OH^−^

3Fe^2+^ + HCrO_4_ ^−^ + 8H_2_O → 3Fe(OH)_3_(s) + Cr(OH)_3_(s) + 5H^+^

An air bubbling-cathode in an air–cathode dual-chamber microbial fuel cell (MFC) can reduce Cr^6+^ efficiently. When Cr^6+^ is reduced in situ at a carbon-felt cathode, the electrogenerated H_2_O_2_ at the cathode driven by iron-reducing bacteria can be strongly associated with the reduction of Cr^6+^ [323]. Enzymatic anaerobic Cr^6+^ reduction involves members of the cytochrome family (e.g., cytochrome b and c) [324].

#### 2.4.3. Cr^6+^ Reducing Fungi

The species of fungi which can be reduced to Cr^6+^ to Cr^3+^ can be identified by a number of processes such as isolation, characterization, and examination of their Cr-reducing capability under various conditions. Two chromate-resistant filamentous fungi, *Aspergillus* sp. N_2_ and *Penicillium* sp. N_3_ were used to test their reduction efficiency in 50 mg/L Cr^6+^ solution at almost neutral pH. *Aspergillus* sp. N_2_ and *Penicillium* sp. N_3_ exhibited 75% and 35% reductions, respectively. The enzymatic reduction and sorption to mycelia were the mechanisms of this method [325]. Gola et al. (2016) investigated the efficiency of *Beauveria bassiana* in the removal of Cr^6+^ [326]. The study found that maximum Cr removal was possible at neutral pH when the temperature was kept at 30 °C at 150 rpm and 120 h. Chakraborty et al. (2018) used *Aspergillus* sp., isolated from soil of the Sunderban mangrove forest, West Bengal, for the efficient removal of Cr^6+^ (98.96%) at pH 4 [327]. Chatterjee et al. (2020) successfully fabricated superparamagnetic iron oxide nanoparticles (IONPs) (Fe_3_O_4_) of 20–40 nm size using manglicolous (mangrove) fungus *Aspergillus niger* BSC-1 for the removal of Cr^6+^ from aqueous solution [328]. Maximum removal of Cr^6+^ occurred at 40 °C, pH 3, and with a 2.5 g/L dose of IONPs. The biological removal of Cr^6+^ is listed together in Table 6.

### 2.5. Membrane Filtration Process

Technological advancements in membrane development have led to an increase in the use of membranes over the years for the removal of Cr from wastewater. There are several factors that can affect membrane separation, including material, membrane pore size, and composition [329]. Various other technologies were also used with membrane technology, including ion exchange, adsorption, and electrochemistry. Among the membrane filtration processes, reverse osmosis is considered to be one of the best available technologies for Cr removal [21,330]. A few publications have favored the nanofiltration process for removing Cr^6+^ [330]. The performance of membranes for the treatment of Cr^6+^ is illustrated in Table 7.

Sulfated carboxymethyl cellulose nanofilter membrane by cross-linking of glutaraldehyde (GA) supported on polysulfone membrane (SCMC-GA-NF) was undertaken by Gasemloo et al. (2019) [333]. The maximum Cr^6+^ removal efficiency of 79.85% was obtained at pH = 4, pressure = 3 bar, and SO_3_/Pyridine:CMC ratio (1:1) in filter. Wu et al. (2018) prepared an efficient adsorbent, nano Uio-66-NH_2_ metal–organic frameworks (MOFs) [334]. The maximum adsorption capacity was 32.36 mg/g at pH 6.5. The process fitted the pseudo-second-order kinetics model and Langmuir isotherm model best. Liu et al. (2019) utilized green tea extract for the biosynthesis of iron nanoparticles (FeNPs)-calcium alginate (CaAlg) hydrogel membrane [335]. The FeNPs-CaAlg hydrogel membrane (0.6 g) can remove as high as 99.5% of 1 mg/L (50 mL) Cr^6+^ at room temperature (23 °C) and original pH (5.41) within 10 min.

Koushkbaghi et al. (2018) prepared dual-layer mixed matrix membranes (MMMs) by incorporating aminated Fe_3_O_4_ nanoparticles into the chitosan/polyvinyl alcohol nanofibers polyethersulfone (PES) membrane for the removal of Cr^6+^ [336]. The maximum adsorption capacity of Cr^6+^ was found to be 509.7 mg/g at optimum pH of 3 in a binary system. Zhijiang et al. (2017) [337] used surface amidoxime-modified polyindole (SAMPI) nanofiber membrane for the removal of Cr^6+^. Maximum adsorption occurred at pH 3 while the other parameters were: temperature 25 °C, initial Cr^6+^ concentration 400 mg/L, and contact time 24 h. The isotherm fitted the Langmuir isotherm model best. Jo et al. (2022) prepared IP-assembled poly(acryloyl hydrazide)-branched star polymer (PAH-TFC) for the removal of hexavalent chromium from wastewater. It exhibited significantly higher Cr^6+^ rejection compared to other representative commercial RO membranes (Cr^6+^ rejection of ∼55% at pH 3) with similar water permeance [338].

Because of its poor removal capacity, the use of MF in Cr removal has not received adequate attention. Nonetheless, it has been utilized by altering the feed solution’s membrane or chemical pre-treatment.

### 2.6. Chelation

Chelation is another method employed for removing Cr. Malek et al. (2009) described a novel strategy that allows chromium-containing leather wastes to be decontaminated [340]. These researchers used two different procedures to remove Cr from wastewater.

Organic salts and acids such as potassium oxalate, potassium tartrate, and acetic and citric acids were tested for their efficiency to separate the chromium from the leather waste.

This ability was determined by the amount of chromium extracted with each organic chelate, and the best yield was about 95%. In their first experiment, they noticed that the effect of organic salts on chromium extraction yield was more important than that of organic acids. Of the different chelates tested, tartrate generated the best results, with approximately 69.7% of chromium extraction yield [340].

Generally, after treatment in a basic medium, the residual chromium content obtained in each case steadily falls to the 0.24–1.1% range. According to the results of both procedures, it is concluded that alkaline medium is the best way and potassium tartrate is the most efficient medium for chromium extraction. In their recent study, Shukla et al. (2022) [341] statistically designed lab-scale soil washing experiments using two chelating agents, i.e., ethylene diamine tetra acetic acid (EDTA) and *N*-acetyl-L-cysteine (NAC). The effects of pH, contact time, and dosage of wash solution on chromium (Cr^+6^) removal efficiency (RE) were investigated and optimized using response surface methodology (RSM). The projected Cr^+6^ RE under these optimum conditions (pH—5.5, contact time—216 h, dosage of EDTA—4128 mg/kg, and dosage of NAC—300 mg/kg) was 14.3% and 65.7%, for EDTA and NAC, respectively. The results of this investigation indicate that washing soil with NAC could be a better alternative to EDTA for removing Cr^+6^ from the soil [341].

The membrane was able to adsorb 99.5% Cr^6+^ at pH 3. A flexible polypyrrole (PPy) membrane with bayberry-like vesicle structures (PPy-B) was prepared by Li et al. (2022) who resorted to template-assisted interfacial polymerization [339]. The PPy-B membrane exhibited an enhanced adsorption capacity of Cr^6+^ (586.90 mg/g) when pH 2 was maintained. The membrane was capable of adsorbing 99.5% Cr^6+^ at pH 3. A flexible PPy membrane with bayberry-like vesicle structures (PPy-B) was prepared by Li et al., (2022) via template-assisted interfacial polymerization [339]. PPy-B membrane exhibited an enhanced adsorption capacity of Cr^6+^ (586.90 mg/g) when pH 2 was maintained.

## 3. Conclusions

Among all the heavy metals, Cr is one of the potentially toxic substances. Because of its toxicity and carcinogenicity, Cr has become an important problem to research; its presence in water and the devised removal technologies have been well documented. In this review, we discussed various Cr removal technologies, for instance, adsorption, electrochemical treatment, physico-chemical process, and biological removal of both Cr^3+^ and Cr^6+^ from water. Cr^6+^ removal was evaluated when different conditions were taken into account, such as pH, initial Cr concentration, temperature, ratios, etc. It would not be a wise decision to restrict the effluent treatment methods to laboratory conditions and synthesized samples alone. Based on the advantages and disadvantages of different methods, the most viable treatment technique should be chosen depending on the initial Cr concentration, operational cost, and wastewater characteristics. However, further research is required on more efficient, cost-effective, and progressively recyclable methods that will produce less noxious byproducts during adsorption. Finally, more research in the future and especially on the systematic application of Cr remediation in the environment is urgently required.

## Figures and Tables

**Table 1:** Advantages and disadvantages of Cr removal methods.

Name of the Technique	Name of Methods	Advantages	Disadvantages	References
Adsorption/Sorption	Adsorption	Both anionic and cationic Cr species can be absorbed Removal of Cr is comparatively higher than precipitation process Efficient to remove both Cr^3+^ and Cr^6+^ Minimum sludge production. Recycle of adsorbent	Adjustment of pH is difficult Limited capacity Requires proper disposal system to reduce environmental pollution	[34,35]
Electro-chemical treatment	Electrocoagulation	Requires minimal capital Requires low pretreatment Both Cr^3+^ and Cr^6+^ can be removed efficiently Cr can be recovered for further use Minimum reaction time Fe electrode is more efficient for Cr^6+^ removal	Multi-stage process required Difficulties may arrive due to the presence of complex metal and anion Difficulties in the removal of micro flocks Huge amount of sludge is produced Polishing treatment may be required for colloidal flocks removal	[36,37,38]
	Electro-floatation	Does not require additional chemical Easy to control Electrode can give total coverage	Big bubbles can hinder the process	[39]
	Electro chemical reduction	Minimum time requires for both reaction and retention Single step process Minimum metal residue concentration	Excess precipitation of iron hydroxide increases sludge quantity	[36,40]
Physico-chemical treatment	Ion exchange method	Easy to operate Highly Effective and reliable Efficient for small and large installation Varieties of resins are available	Regular regeneration Concentrate disposal Resin fouling Other ions can interfere with removal efficiency	[41,42,43,44,45]
	Reduction process	Single step process Low concentration of metal residue Reductants can be dehydrated readily	Costly and unsuitable for drinking water treatment	
Biological removal		Eco friendly Simple operation	Time-consuming process	[6]
Membrane filtration	Reverse osmosis	One of the best available technology for Cr removal 60–100% removal is possible Wide range pollutants can be removed effectively and simultaneously	High investment and operational cost Membrane fouling Disposal of brine	[38,46]
	Nano filter	Has bulk surface area	Requires pre-treatment	

**Table 2:** Types of adsorbents and their maximum Cr removal capacities. [Superscripts a, b, and c indicating removal amount in percentages, mg/L, and μmol g^−1^, respectively. L = Langmuir, F = Freundlich, T = Toth, R–P = Redlich–Peterson, S = Sips, E = Elovich, D–R = Dubinin–Radushkevich, D = Dahlquist, S = Scatchard, Tm = Temkin].

Type of Adsorbent	Adsorbent	pH	Removal Quantity (mg/g)	Isotherm	References
Agricultural Wastes	Bagasse sugarcane	2.0	13.4	L, F	[62]
	Bran rice	2.0	285.7	L, F	[63]
	Wheat bran	2.1	35	L	[64,65]
	Olive cake	2.0	35.44	L, F	[66]
	Sawdust Acacia arabica (pretreated)	6.0	111.6	L, F	[67]
	Shell Almond	4.0	22.20	L, F	[68]
	Lignin from paper Industry	3.0	13.48	L	[69]
	Soya cake	<1.0	0.28	L, F	[70]
	Coir Coconut	2.0	6.3	L, F, R–P, T, etc.	[71]
	Husk and hull Bengal gram husk	2.0	91.64	L, F	[72]
	Acrylonitrile grafted banana peels	3.0	96 ^a^	L, F	[73]
	Rice husk	5.0	38.4	-	[74]
	Rye husk	3.0	80 ^a^	L	[75]
	Chitosan-coated banana and areca fiber	4.5	75 ^a^	-	[76]
	Rice husk	6	92.99 ^a^	L	[77]
	Saw dust	2	100 ^a^	F	[78]
	SCM-CC	2	99.92 ^a^	F	[79]
	Green walnut shell	3.6	95 ^a^	-	[61]
Plants	Sugarcane bagasse	1.9	23	L, D, S	[80]
	Sunflower stem waste (pretreated)	2.0	5.37–4.81	L, F, D-R	[81]
	London plane leaves	3.0	68.03	L	[82]
	Modified Pine sawdust	2.0	30.49	L, F	[83]
	Hazelnut shell	3.5	8.28	L, F	[84]
	Almond shell	2.0	10.61	L, F	[66]
	Rubber wood sawdust (Polyacrylamide grafted)	3.0	22.6	F	[85]
	*Tradescantia pallida* leaf	2.0	94 ^a^	L	[86]
	*Caryota urens* seeds	2.0	52.63	L	[87]
	Aminated rice straw-grafted-poly (vinyl alcohol) (A-RS/PVA)	2.0	140.39	E, F	[88]
	Paddy straw	2.0	21.50	L, D, S	[89]
	Rubber leaf	1.5	96 ^a^	-	[58]
Algae	*Scenedesmus* sp.	6.0	98.63 ^a^	-	[90]
	*Scenedesmus quadri-cauda* (Chlorophyta)	6.0	98.3 ^a^	L, F	[91]
	*Sargassum myriocystum*	5.2	66.66	L, T	[92]
	*Cladophora glomerata*	2.0	66.6	L, F	[93]
	*Oedogonium hatei*	2.0	35.2	L	[94]
	*Sargassum sp*	2.0	39.61	L	[95]
	*Nostoc calcicola HH-12*	3.0–4.0	12.23	L	[96]
	*Chroococcus* sp. *HH-11*	3.0–4.0	21.36	L	[96]
	*Sargassum seaweed (marine algae)*	3.5	60 ^b^	L, F	[97]
	*Scenedesmus incrassalulus (green micro algae)*	-	52.7 ^a^	-	[98]
	*Spyrogyra species (green filamentous algae)*	2.0	90 ^a^	L	[99]
Fungi	*Penicillium janthinellum*	1.0	58.6 ^a^	F, D-R	[100]
	*Aspergillus niger*	2.0	11.79	Tm, F	[101]
	*Aspergillus niger*	3.0	95.7 ^a^	-	[102]
	*Aspergillus ustus*	2.0	6466.7 ^c^	L, F	[103]
	*Fusarium verticillioides*	2.0	6400.0 ^c^	L, F	[103]
	*Pencillium funiculosum*	2.0	3800.0 ^c^	L, F	[103]
	*Aspergillus niger*	3.0	96.3 ^a^	-	[104]
	*Agaricus bisporus*	1.0	8.0	F	[105]
	*Aspergillus niger*	2.0	17.92	F	[106]
	*Aspergillus sydoni*	2.0	9.07	L	[106]
	Marine *Aspergillus niger*	1.0	117.33	L	[107]
	*Basidiomycete,* BDT-14	6.5	83.33	L	[108]
	*Aspergillus* sp. (filamentous)	2.0	10.0–27.5	L, F	[109]
	*Fusarium* sp. (filamentous)	5.0	18.2–71.0	-	[110]
Bacteria	*Spirulina* sp.	5.0	90.91	F	[111]
	*Eshcherichia coli* and *Staphylococcus epidermidis*	3.0–6.0	16.9	L, F	[112]
	*Rhodococcus opacus*	5	82	F	[113]
	*Rhodococcus rhodochrous*	5	62	F	[113]
	*Staphylococcus* sp. and *Pseudomonas* sp.	5		L, F	[114]
	*Azotobacter beijreinckii*	-	26 ^a^	-	[115]
	*Bacillus subtilis*	-	48 ^a^	-	[115]
	*Bacillus licheniformis*	2.5	60.5	L	[116]
	*Bacillus subtilis*	2.0	14.54	L	[117]
	*Staphylococcus xylosus*	1.0	143	L, F	[118]
	*Pseudomonas* sp.	4.0	95	L, F	[118]
	*Rhodococcus opacus*	6.0	72.9	L	[119]
	*Streptomyces rimosus*	4.8	83	L	[120]
	*Bacillus circulans* biofilm	7.0	48 ^a^	-	[121]
	*Bacillus circulan*	2.5	34.5	-	[122]
	*Bacillus megaterium*	2.5	32	-	[122]
	*Bacillus coaglans*	2.5	23.8	-	[123]
	*Microbacterium liquuefaciens MP30*	-	90 ^a^	-	[124]
Activated Carbon	*Aegle marmelos* fruit shell	2.0	82.3 ^a^	-	[59]
	Animal bone charcoal	2	92 ^a^	-	[60]
	Cellulose-clay composite	4	2.37	-	[125]
	CHA/MFC	-	2.208 ^d^	-	[126]
	CNC/clay composite	4	100 ^a^	-	[127]
	Cellulose/chitosan composite	4	56 ^a^	-	[128]
	Clay-alumina ceramic membrane	-	91.44 ^a^	-	[129]
	MWCNTs-CTAB	5	98 ^a^	L	[130]
	MWCNTs-M-SLS	5	99 ^a^	L	[130]
	Chi@Fe_3_O_4_	-	142.32	-	[131]
	Chi@Fe_3_O_4_GO	-	100.51	-	[131]
	FeNi@HPC	4	30 ^b^	-	[132]
Miscellaneous	Shale waste rock	3–6	90–91 ^a^	L, F	[133]
	Iron/biochar beads (FMIB)	4	87.7 ^a^	L	[134]
	Zn and Al modified pristine hydrochar	2–4	65 ^a^ (Zn-HC) 50 ^a^ (Al-HC)	L	[135]
	CDGF	3	99.8 ^a^	-	[136]
	m-phenylenediamine-modified magnetic chitosan	<4	227.27	-	[137]
	TWNP	3.0	59.88 (64 ^a^)	L	[53]
	HFCM	4.0	40 ^a^	-	[57]
	Brown clay	4.0	90 ^a^	-	[54]
	Clinoptilolite	4.0	85 ^a^	-	[56]

**Table 3:** Cr removal capacities of available adsorbents in the literature. (The superscripts a and b represent the results in percentages (%) and mg/L).

Adsorbents	pH	Removing Capacities (mg/g)	References
Iron (nanoparticles)	7	-	[152]
Maghemite (10 nm)	2.5	-	[153]
Akaganeite (3−6 nm)	5.5	80	[154]
MnFe_2_O_4_ (10 nm)	2	31.5	[155]
Carbon nanotube-supported ceria (6 nm)	3.0–7.4	30.2	[153]
δ-FeOOH coated maghemite γ-Fe_2_O_3_ (15 nm)	2.5	25.8	[156]
Activated charcoal cloth	1	5 mmol/g	[157]
Coconut shell	2–6	75.0–107.1	[158]
Wood	2–6	71.6–87.6	[158]
Dust coal	2–6	60.5–101.9	[158]
Sawdust and waste tires (0.38 mm)	2	30–43	[159]
Cactus	2	8.5–34.5	[66]
Wool	2	8.5–34.5	[66]
Charcoal	2	8.5–34.5	[66]
Pine needles	2	8.5–34.5	[66]
Hazelnut Shell	2.5–3.5	17.7	[160]
Single-walled Carbon Nanotubes (SWCNT)	2.5	2.35	[161]
Multi-walled Carbon Nanotubes (MWCNT)	2.5	1.26	[161]
Unfunctionalized MWCNT	6	98 ^a^	[162]
Oxidized MWCNT	2.05	4.26	[163]
MWCNTs/nano iron oxide	5–6	-	[164]
Modified MWCNT	7		[165]
Activated carbon-coated CNT	4	9.0	[166]
Waste eggshell	6–12	90 ^a^	[167]
Chitosan-coated acid-treated seed shells	4.5–5.0	60–85	[168]
Green algae (*Ulva lactuca*)	1	10.61	[169]
Activated carbon from green algae (*Ulva lactuca*)	1	112.36	[169]
Biofilm of *E. coli* supported on NaY zeolite	4.6–5.1	>85.5 ^a^	[170]
*Pseudomonas aeruuginosa* immobilized MWCNT	8.5–9.5	6.23	[171]
*Saccharomyces carlsbergensis* immobilized on amberlite	8	95 ^a^	[172]
Wallnut shell	3.5	8.01	[84]
Hazelnut shell	3.5	8.28	[84]
Almond shell	3.2	3.40	[84]
*Ficus carica* biosorbent	1.0–3.0	19.68	[173]
Straw from *Triticum aestivum*	5	21	[174]
Wheat Bran from *Triticum aestivum*	>4	35	[64]
Wheat Bran from *Triticum aestivum*	1	40.8	[175]
Wheat Bran from *Triticum aestivum*	2	310.58	[176]
Wheat Bran from *Triticum aestivum*	2	0.942	[177]
Wheat Bran from *Triticum aestivum*	5	93	[178]
Coconut coir	1–5	26.6–27	[71]
Sawdust	3	1.48	[179]
Rice husks	3	0.63	[179]
Coirpith	3	0.16	[179]
Vermiculite	3	0.26	[179]
*Spirogyra condensata*	5	14	[96]
*Rhizoclonium hieroglyphicum*	4	11.81	[96]
*Chlorella vulgaris*	1–5	2.98	[180]
*Clodophara crispata*	1–2	6.20	[180]
*Zoogloea ramigera*	1–2	3.40	[180]
*Rhizopus arrhizus*	1–2	8.40	[180]
*Saccharomyces cerevisiae*	1–2	4.30	[180]
Chitosan-ceramic alumina composite	4	153.85	[181]
Surfactant-modified coconut coir pith	2	76.3	[182]
Cross-linked xanthated chitosan beds (CMBC)	3	256.4	[183]
Cross-linked xanthated chitosan flakes (CMCF)	3	625	[183]
Eggshells	3.54	81.47 ^a^	[184]
Carrot residue	4	45.09	[185]
Fungal biomass	2	119.2	[186]
Chitosan-coated fly ash	5	33.27	[187]
Grape waste	4	1.91 mol/kg	[188]
*Dundiella* algae	2	45.5	[189]
Pine Needles powder	3	40	[190]
*Laminaria japonica*	1	96.31	[175]
*P. yezoensis Ueda*	1	95.81	[175]
Rice bran	1	95.35	[175]
PAN-CNTs-TiO_2_-NH_2_ composite	2	80 ^a^	[191]
Hydrophobic magnetic adsorbent based on polypyrrole coating on acid-dissolved fly ash (MSFA/PPy)	-	66.93−119.33	[151]
MWCNTs-COOH	2	143–164	[192]
LDHs@MoS_2_	5	76.3	[193]
Shrimp shell and waste cotton rags	5	93 ^a^	[138]
PANI-NC	6	92.59	[194]
FeNi@HPC	4	30 ^b^	[132]
Cellulose and chitosan composite	4	60 ^b^	[128]
Red mud and rice straw	6	97.74 ^a^	[195]
Carbonized chitosan into triethylenetetramine-modified sodium alginate (CTS/CS-50)	1	144.49	[196]
nZVI/ZIF-8	5	>99 ^a^	[197]

**Table 4:** Removal of Cr by electrocoagulation (EC).

Types of Electrode	pH	Removal (%)	Current Density	References
Fe-S 304	6.9	97	50 A m^−2^	[233]
Al-S 304	5	15	50 A m^−2^	[233]
Fe-Al	3	100	10 mA cm^−2^	[234]
Fe-Fe	9.56	100	4 mA cm^−2^	[235]
Mild S	5.91	99	1000 mA	[236]
Al-Fe	4	99	-	[237]
Al alloy-galvanized Fe	7	98.2	0.2 A dm^−2^	[238]
Fe-Fe, Al-Al, Al, Pt, Ti, Pt/Ti/Fe,		<0.5 mg/L	1 Am^−2^	[239]
Fe-Al	7–9	>90	2 mA cm^−2^	[240]
Fe-Fe	4	100	50 mA cm^−2^	[241]
Fe-Al	2	100	0.73 mA cm^−2^	[242]
Al-Al/Cu/Mg alloy	5.3	99	400 Am^−2^	[243]
Al-Al	3.5–4.0	90–99.8	11.57 Am^−2^	[244]

**Table 5:** Photocatalytic degradation and reduction of available used catalysts in the literature.

Electron Donor	Type/Source of Catalyst	Reduction (%)	Contact Time	Radiation	References
Tartaric, citric, malic, and n-butyric acids	Diluted and Fe(III) adsorbed onto clay	100	7–80 min	Visible Light	[289]
Tartaric and citric acids	Soils	100	4 h	Mimic Solar	[299]
Alginate	γ-Fe_2_O_3_ nanoparticles	≈100	50 min	Sunlight	[300]
Salicylic acid	CuFe_2_O_4_ nanoparticles	60	2.8 h	-	[301]
	TiO_2_ powder	95	2 h	visible light	[302]
-	TiO_2_ nanoparticles modified with C_60_(CHCOOH)_2_	97	1.5 h	UV radiation	[303]
-	La_2_Ti_2_O_7_ and salts (NaCl, KCl, CaCl_2_, MgCl_2_, Na_2_SO_4_)	98	3 h	UV light	[304]
Salicylic acid	TiO_2_ powder	-	300–900 min	UV 253.7 nm	[305]
Methanol, formic acid, acetic acid, triethanolamine, EDTA	TiO_2_	100	-	550 nm, visible light	[306]
Salicylic acid	CuAl_2_O_4_/TiO_2_	95	3 h	visible light, 1.7–2.5 eV	[307]
Citric acid	WO dopped TiO_2_ nanotube	-	-	UV light	[308]
Other photocatalysts Added electron donor	NiO nanoparticles	90	75 min	Laser Radiation	[309]
Added electron donor	ZnO nanoparticles	95	60 min	Laser Radiation	[310]
	Ag/Ag_3_PO_4_/reduced graphene oxide microspheres	90	30 h	Visible light	[295]
	Mn_3_O_4_@ZnO	95.3	110 min	Sunlight	[296]
	UiO-66-NH_2_(Zr/Hf)	94	120 min	Visible light	[297]
	ZnS-Ga_2_S_3_-3	99.1	160 min	Solar light	[298]

**Table 6:** Biological removal of Cr^6+^ in different literature (the superscript “a” denotes in mg/g).

Species	Initial Conc. of Cr^6+^	pH	Temperature (°C)	Period (Hours)	Reduction (%)	References
*Pseudomonas brassicacearum* LZ-4	10 ^a^	7.5	37	-	80	[316]
*Brevibacterium casei*	5 ^b^	7	35	-	83.4 ± 0.6	[315]
*Bacillus* sp. JDM-2-1	100 ^a^	7	37	96	85	[313]
*Staphylococcus capitis*	100 ^a^	7	37	96	81	[313]
*Pseudomonas aeruginosa* strain G12	10 ^a^	7	30	72	93	[317]
*Pannonibacter phragmitetus*	1000 ^a^	9	37	24	100	[318]
*Pannonibacter phragmitetus* LSSE-09 coated with polyethylenimine-functionalized magnetic nanoparticles	350 ^a^	9	37	0.33	100	[319]
Resting *Escherichia coli* cells	150 ^a^	7	-	4	97.5	[322]
*Bacillus subtilis*	50 ^a^	9	30	65	100	[324]
*Beauveria bassiana*	30 ^a^	7	30	12	61.1	[326]
*Aspergillus* sp.	50 ^a^	4	27	-	98.96	[327]
Iron oxide nanoparticles fabricated *Aspergillus niger* BSC-1	10 ^a^	3	40	2	99.75	[328]

**Table 7:** The performances of membranes for the treatment of Cr^6+^ (the superscript “a” denotes in mg/g).

Initial Concentration (mg/L)	Application/Membrane Type	Pressure (bar)	pH	Removal %	References
0.4	Nanofilter	14	7.1	95	[331]
0.4	Nanofilter	5	7.1	85	[331]
0.1	Nanofilter	5	7.1	52.7	[331]
0.05	Reverse osmosis	100	N. A.	98.3	[332]
0.05	Reverse osmosis	500	N. A.	100	[332]
0.05	Reverse osmosis	3.5	3	99.01	[332]
0.05	Reverse osmosis	3.5	9	>99.9	[332]
10,000	Reverse osmosis	200 psi	6–7	100	[32]
SCMC-GA-NF	Nanofilter	3	4	79.85	[333]
Nano Uio-66-NH2	Nanofilter	-	6.5	32.36 ^a^	[334]
FeNPs-CaAlg	Hydrogel		5.41	99.5	[335]
Aminated-Fe_3_O_4_ chitosan/polyvinyl alcohol-PES	Nanofilter	-	3	509.7 ^a^	[336]
SAMPI	-	-	3		[337]
PAH-TFC	-	-	3	99.5	[338]
PPy-B	-	-	2	586.9 ^a^	[339]

## Data Availability

The data of this study will be shared upon reasonable request to the corresponding author.
